# Interfacial Regulation–Driven Dual‐Enrichment SERS Coupled With Deep Learning Enable Ultrasensitive and In Situ Identification of Microplastics in Natural Waters

**DOI:** 10.1002/smll.74231

**Published:** 2026-06-19

**Authors:** Chaochao Ma, Xiaojiao Zhao, Jiacheng Li, Xinyu Liu, Mingxu Zhang, Zhidan Liu, Jing Wu, Zhifeng Liu, Yunpeng Wang, Yang Li

**Affiliations:** ^1^ State Key Laboratory of Frigid Zone Cardiovascular Diseases (SKLFZCD) Research Center for Innovative Technology of Pharmaceutical Analysis College of Pharmacy Harbin Medical University Harbin Heilongjiang China; ^2^ School of Physical Science and Technology Nantong University Nantong Jiangsu China; ^3^ Inpatient Department Heilongjiang Provincial Eye Hospital Harbin Heilongjiang China; ^4^ Research Unit of Health Sciences and Technology (HST) Faculty of Medicine University of Oulu Oulu Finland; ^5^ Heilongjiang Provincial Clinical Medical Research Center For Chemical Poisoning Heilongjiang Second Provincial Hospital Harbin Heilongjiang China

**Keywords:** in situ environmental monitoring, machine learning, microplastics, SERS, spectral database

## Abstract

Accurate detection of trace‐level microplastics in natural waters remains challenging due to inefficient particle enrichment, unstable particle‐substrate interactions, inhomogeneous hotspot distributions, and spectral similarity among polymers. Here, we develop a membrane‐confined SERS platform integrated with machine‐learning‐assisted spectral analysis for sensitive and reproducible microplastic detection. In this platform, PSS‐Na‐modified Au@Ag nanocubes are employed as plasmonic building blocks to construct a charge‐regulated, vertically stacked hotspot architecture on a cellulose acetate membrane. By improving microplastic enrichment, hotspot accessibility, and interlayer electromagnetic coupling, the membrane‐guided configuration enables reproducible detection of polystyrene down to 50 ng mL^−^^1^. It also delivers highly reproducible signals in microplastic spike samples prepared in six environmental water matrices without chemical pretreatment. To resolve spectral similarity among polymers, a multi‐polymer SERS library covering PS, PMMA, PVC, and PC was integrated with an attention‐based 1D‐CNN, enabling reliable polymer identification and semi‐quantitative analysis of binary and ternary mixtures (R^2^ > 0.83). This membrane‐confined plasmonic platform, together with data‐driven spectral analysis, provides a robust route for intelligent microplastic monitoring in complex aquatic environments.

## Graphical abstract

A membrane‐confined SERS platform constructs a dense network of vertical hotspots using PSS‐Na‐modified Au@Ag nanocubes for sensitive and reproducible microplastic detection in complex environmental water matrices. Coupled with an attention‐based 1D‐CNN, it enables accurate polymer identification and semi‐quantitative mixture prediction for intelligent microplastic monitoring.

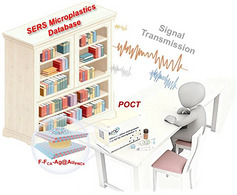

## 1 Introduction

The widespread production and use of plastics have greatly benefited modern society since the 1950s, but the accumulation of plastic waste has become a major global environmental problem. A substantial fraction of discarded plastics accumulates in landfills or leaks into the natural environment, where they slowly break down into microplastics (MPs, 1 µm–5 mm) and nanoplastics (NPs, <1 µm) due to the effects of light, heat, mechanical forces, and biological processes [1, 2, 3]. These particles are highly stable and mobile, and have been found in a variety of environmental media and biological tissues. Increasing evidence suggests that these particles may pose ecological and health risks, including inflammation, metabolic disorders, and reproductive toxicity [4, 5]. The hidden nature and complexity of microplastic contamination make its detection a critical challenge in environmental science [6, 7]. Current mainstream detection methods include pyrolysis–gas chromatography/mass spectrometry (Py‐GC/MS) [8, 9], electron microscopy (SEM, TEM) [10, 11], and Fourier transform infrared spectroscopy (FT‐IR) [12, 13]. Nonetheless, these methods often require complex sample preparation, dedicated instrumentation, or destructive analysis, and are not well suited for fast field screening. Therefore, there is an urgent need to develop a sensitive, non‐destructive, easy‐to‐use platform for rapid and reliable microplastic detection in complex aquatic environments.

Surface‐enhanced Raman spectroscopy (SERS) has emerged as a promising technique for environmental analysis due to its high sensitivity, label‐free detection, and molecular specificity. However, its application to microplastic detection remains challenging. First, the similar chemical structure of different microplastics results in overlapping Raman peaks, limiting spectral discrimination. Second, the particulate nature of microplastics limits effective contact with plasmonic hotspots and prevents stable electromagnetic coupling, leading to large signal fluctuations and poor reproducibility. It is important to note that, unlike small‐molecule detection, the key limitation in granular microplastic systems lies not only in the signal strength but also in the instability of the particle‐substrate interfacial interactions. To address these issues, researchers have focused on improving substrate design and signal enhancement methods. For example, Pant et al. [14], used gold nanoparticles with Al/Cu foil to create SERS substrates that effectively boosted polystyrene (PS) signal strength; Zhang et al. [15] increased SERS signals of PET and PS in bottled water using triangular cavity array substrates; Kim et al. [16], visualized PS and PE nanoplastics through Raman imaging. Zhao et al. [17], developed a “sandwich” liquid–liquid interface structure to increase hotspot density and signal consistency. While these strategies have improved local electromagnetic enhancement and detection sensitivity, challenges such as complex fabrication, limited structural stability, and insufficient adaptability to the environmental matrix remain. More importantly, most existing approaches mainly focus on increasing the field strength, while the regulation of interfacial interactions and signal reproducibility in granular microplastic systems remains under‐explored. At the same time, algorithm‐driven spectral identification methods are demonstrating growing potential. Xie et al. [18], achieved high‐precision classification of nanoplastics (98.8% accuracy) using a random forest (RF) model, confirming machine learning's feasibility for SERS spectral discrimination. However, many existing models still rely on shallow learning frameworks and may show limited robustness against signal fluctuations, spectral similarity, and matrix interference in complex environments. Therefore, a SERS platform that simultaneously addresses interfacial stability, signal reproducibility, and intelligent spectral analysis is highly desirable.

**FIGURE 1:**
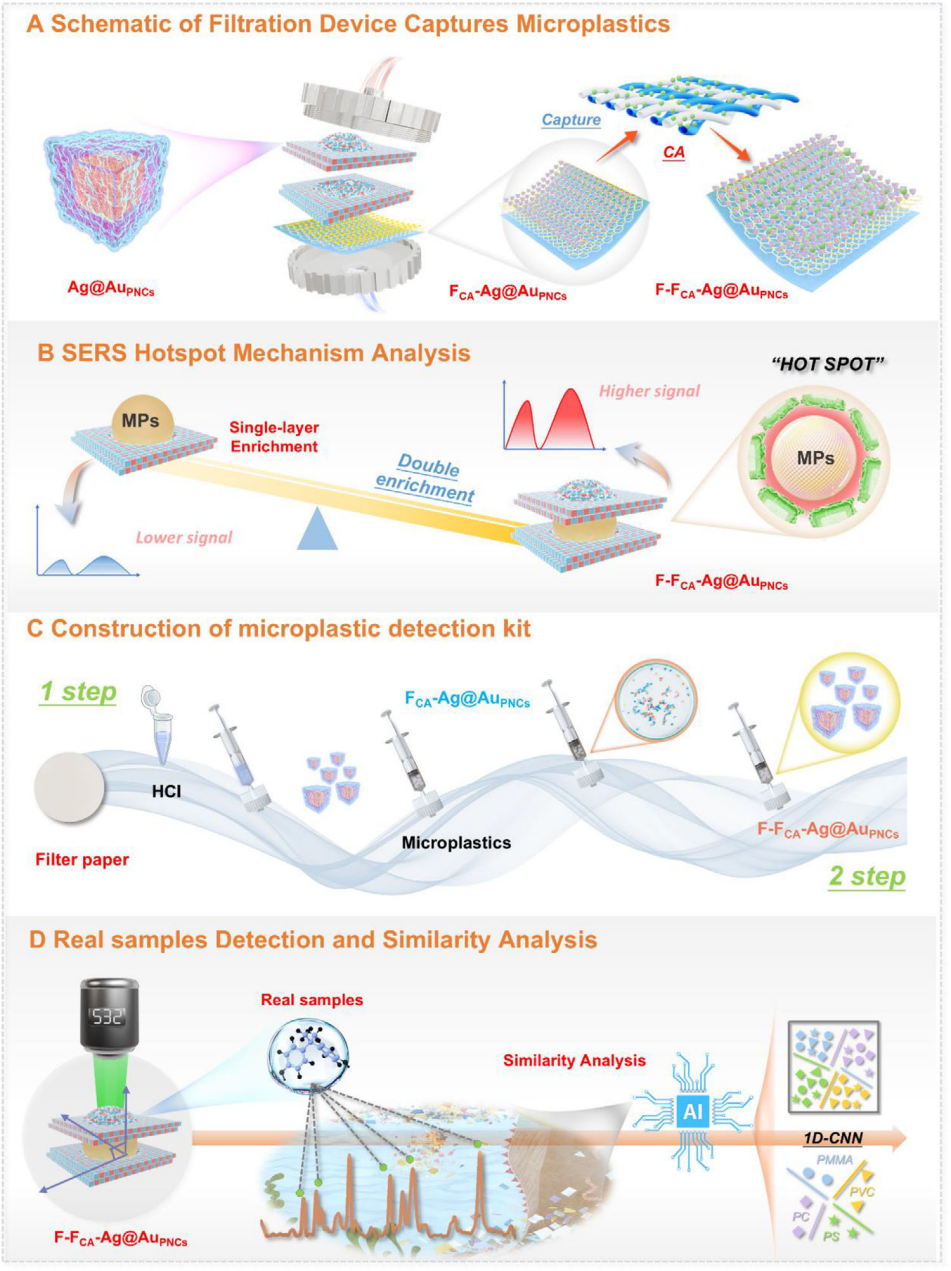
Schematic of the preparation, application, and mechanism of the programmable SERS microplastic detection kit. (A) Schematic of Filtration Device Captures Microplastics. (B) SERS Hotspot Mechanism Analysis. (C) Schematic of the detection process and mechanism of the microplastic detection kit. (D) Detection of microplastics in real water systems and database‐driven similarity recognition. This schematic illustrates the kit's overall design philosophy and innovative features: a dual‐enrichment strategy achieves efficient enhancement of microplastic SERS hotspots, while database‐driven similarity analysis algorithms overcome signal overlap and poor reproducibility challenges, enabling high‐precision detection and intelligent identification of multiple microplastic types in complex aquatic environments.

In this study, we develop a membrane‐confined SERS platform integrated with machine‐learning‐assisted spectral analysis for sensitive and reproducible microplastic detection. Unlike our previous Au@Ag_PNC_‐based hybrid substrate for molecular sensing [19], the present work uses Au@Ag_PNCs_ mainly as plasmonic building blocks and focuses on membrane‐guided microplastic enrichment, vertical hotspot construction, and machine‐learning‐assisted spectral recognition. Specifically, we propose a membrane‐based strategy in which charge‐regulated nanoparticle assembly is used to improve nanoparticle–microplastic contact and hotspot homogeneity (workflow shown in Figure 1). The membrane‐confined architecture promotes microplastic enrichment and hotspot accessibility, while the bilayer nanoparticle structure facilitates vertical electromagnetic coupling relative to a planar monolayer configuration. To address the spectral similarity among polymers, we further build a multi‐polymer SERS spectral library and implement an attention‐based 1D‐CNN model for intelligent spectral identification. By integrating membrane‐confined microplastic enrichment, bilayer hotspot engineering, and machine‐learning‐assisted spectral analysis, this work provides a unified strategy for sensitive, reproducible, and robust microplastic detection in complex aquatic environments.

## 2 Results and Discussion

### 2.1 Charge‐Regulated Interfacial Assembly of Au@Ag_PNCs_ With Microplastics

**FIGURE 2:**
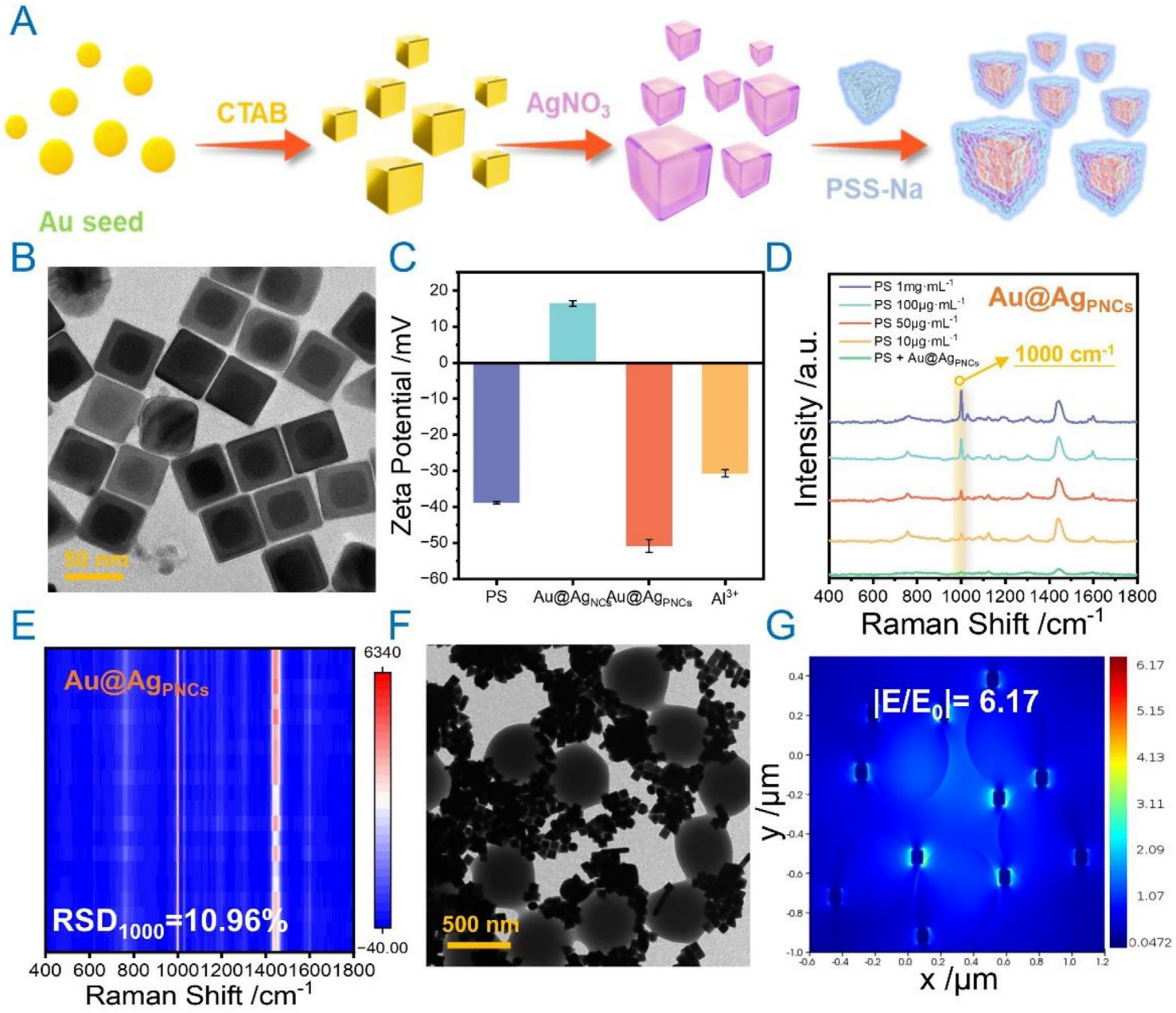
Schematic illustration of the synthesis, characterization, and performance of silver‐coated gold nanocubes (Au@Ag_NCs_) in microplastic detection. (A) Schematic of synthesis and surface modification of PSS‐Na modified silver coated gold nanocubes (Au@Ag_PNCs_). (B) TEM image of Au@Ag_NCs_. (C) Zeta potential distribution of PS and Au@Ag_NCs_, revealing electrostatic adsorption. Potential changes of PSS‐Na modified Au@Ag_PNCs_ before and after Al^3+^ addition (*n* = 3, means ± SD). (D) SERS spectra of PS microplastics detected by PSS‐Na‐modified Au@Ag_PNCs_ under different conditions. The curve labeled “PS + Au@Ag_PNCs_” corresponds to the SERS spectrum of PS (1 mg mL^−1^) obtained without the addition of Al^3+^ as an aggregation agent. (E) Stability evaluation of Au@Ag_PNCs_ detection signals (*n* = 50). (F) TEM image after Al^3+^ addition, showing significantly enhanced hotspot density. (G) FDTD simulated electric field distribution revealing strong localized electromagnetic coupling effects. Overall, results indicate this system follows a multiple‐level enhancement mechanism of “charge regulation, ionic bridging and electromagnetic synergy”: PSS‐Na improves particle dispersion and interfacial stability, while Al^3+^ bridging enhances hotspot coupling, achieving highly reproducible and programmable SERS enhancement.

Au@Ag core–shell nanocubes (Au@Ag_NCs_) were employed as structurally defined plasmonic building blocks to investigate charge‐regulated nanoparticle–microplastic interfacial assembly (Figure 2A). TEM and SEM images confirmed the uniform cubic morphology of Au@Ag_NCs_, while elemental mapping verified the typical Au‐core/Ag‐shell structure (Figure 2B; Figures S1 and S2). Additional characterization, including HRTEM, DLS, XRD, and UV–vis spectroscopy, is provided in the Supporting Information (Text S1 and Figures S3–S5), as these results mainly serve to confirm the crystallinity, size distribution, and optical response of the nanocubes. Together, these results confirm that Au@Ag_NCs_ are suitable as reproducible plasmonic building blocks, consistent with our previous report [19]. The following discussion therefore focuses on the charge‐regulated nanoparticle‐microplastic interfacial assembly and its translation into a membrane‐confined SERS detection platform.

The as‐prepared CTAC‐stabilized Au@Ag_NCs_ exhibited a pronounced SERS response toward PS microplastics, with a characteristic band at 1000 cm^−1^ assigned to the aromatic ring‐breathing vibration of the phenyl group in polystyrene. However, signal reproducibility was poor, with a lowest detectable concentration of 50 µg mL^−1^ (S/N > 3, RSD = 23.98%, Figures S6 and S7). This result indicates that strong plasmonic activity alone is insufficient for reproducible detection of particulate microplastics. TEM observations show heterogeneous adsorption of CTAC‐stabilized Au@Ag_NCs_ on PS surfaces, suggesting that the poor reproducibility originates mainly from uncontrolled nanoparticle‐microplastic interfacial assembly (Figure S8). Zeta potential measurements show that PS exhibits a surface potential of −38.80 ± 0.36 mV, whereas the CTAC‐stabilized Au@Ag_NCs_ display a positive potential of +16.40 ± 0.80 mV (Figure 2C). While this electrostatic attraction favors nanoparticle adsorption, the absence of spatial regulation may lead to stochastic aggregation, heterogeneous hotspot formation, and unstable SERS response. In addition, the residual CTAC generates a significant Raman background signal and may hinder efficient contact between PS particles and plasmonic hotspots, further reducing the spectral stability.

To improve interfacial control, CTAC was replaced by PSS‐Na, yielding negatively charged PSS‐Na‐modified Au@Ag_NCs_, denoted as Au@Ag_PNCs_, with improved colloidal dispersion [20].

The disappearance of the CTAC‐dependent Raman band and the reversal of the zeta potential confirm successful ligand exchange and improved colloidal stability (Figure S9; Figure 2C). Since both PS particles and Au@Ag_PNCs_ were negatively charged after PSS‐Na modification, Al^3+^ was introduced to mediate interfacial assembly through multivalent electrostatic bridging, charge screening, and possible coordination with anionic groups [21, 22]. Compared with monovalent or divalent ions, including Na^+^, Mg^2+^, Ca^2+^, and Zn^2+^ (Figure S10), Al^3+^ reduced the lowest detectable concentration to 10 µg mL^−1^ (S/N > 3, Figure 2D). Importantly, this charge regulation strategy does not directly tune the intrinsic LSPR of individual Au@Ag_PNCs_. Instead, it modulates the nanoparticle adsorption, interparticle spacing, and assembly configuration at the PS surface, thereby modulating the interparticle plasmonic coupling. SERS mapping heatmaps reveal a more homogeneous signal distribution with a reduced RSD of 10.96% at 1000 cm^−1^ (Figure 2E), while TEM observations show denser and more interconnected nanoparticle domains on the PS surface (Figure 2F), supporting the formation of a more uniform hotspot network. DLS measurements further supported the Al^3^^+^‐mediated interfacial assembly process. The hydrodynamic diameter of Au@Ag_PNCs_ was 74.19 ± 0.42 nm, while that of bare PS microplastics was 504.50 ± 3.10 nm. After Al^3^^+^‐mediated assembly with Au@Ag_PNCs_, the apparent hydrodynamic diameter of the PS/Au@Ag_PNCs_ assemblies increased to 540.77 ± 12.57 nm. This increase, relative to bare PS microplastics, supports the formation of Au@Ag_PNCs_ assemblies on PS microplastics via ion‐mediated interfacial bridging (Figure S11). FDTD simulations further reveal localized electromagnetic field enhancement at interparticle junctions, with |E/E_0_| reaching 6.17, supporting the presence of assembly‐dependent hotspots and their contribution to the observed SERS response (Figure 2G).

Overall, this section demonstrates that Au@Ag_PNCs_ function as charge‐regulated plasmonic building blocks for microplastic detection. The enhanced SERS performance arises mainly from regulated nanoparticle–microplastic interfacial assembly, rather than from uncontrolled aggregation or intrinsic nanocube properties alone [23, 24, 25, 26]. This interfacial assembly principle provides the mechanistic basis for the membrane‐confined bilayer SERS platform described in the following section, where microplastic enrichment, hotspot accessibility, and vertical electromagnetic coupling are further integrated to enable sensitive and reproducible detection.

### 2.2 Membrane‐Confined Bilayer Hotspot Platform for Microplastic Enrichment and Detection

**FIGURE 3:**
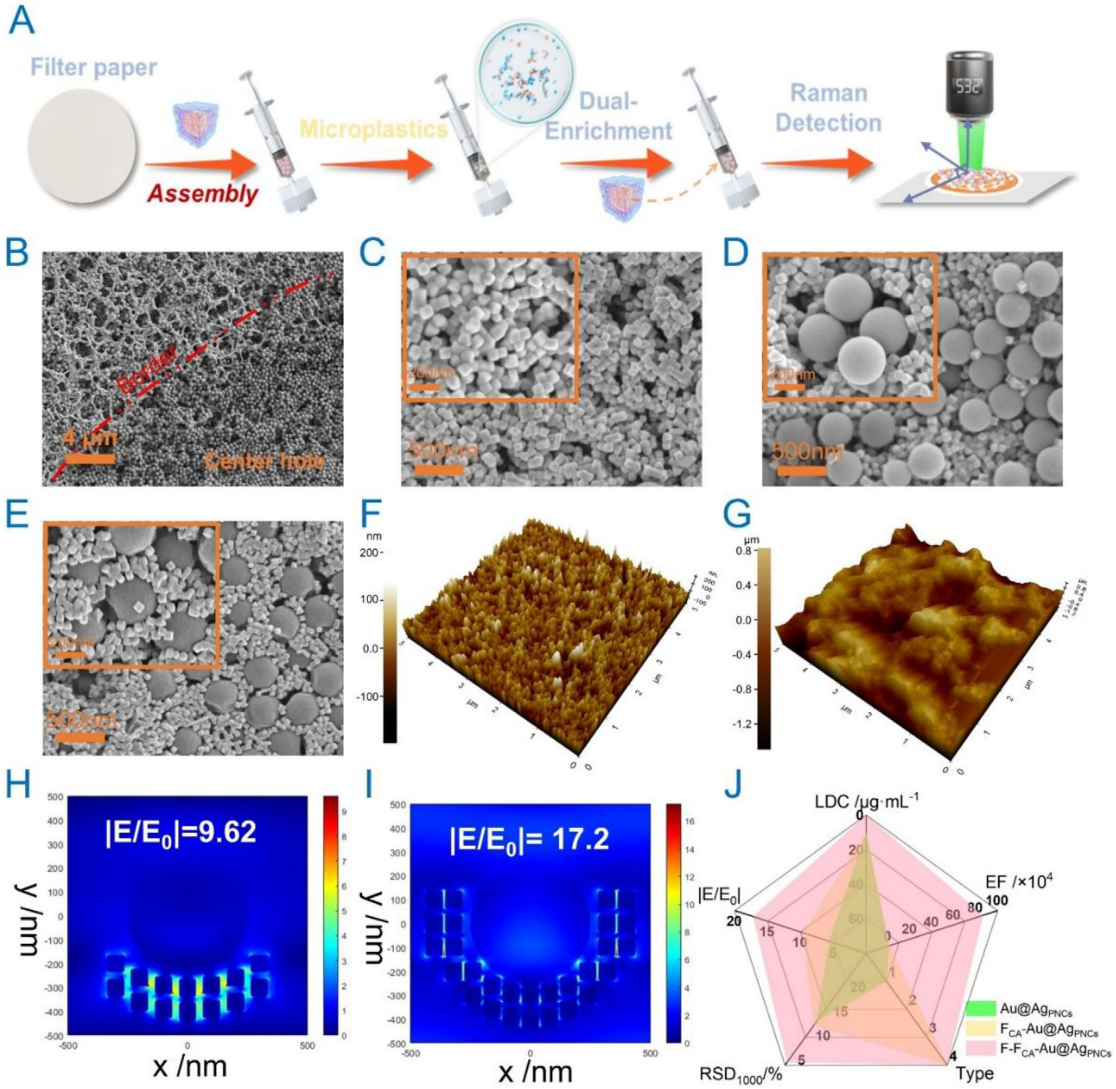
Schematic of microplastic detection process and optimized workflow. (A) Schematic of assembly and detection process for the novel microplastic detection kit. (B) SEM image of microplastics filtered onto CA filter paper, with dashed red lines indicating boundaries, showing nanoparticle concentration at the syringe's central orifice. (C) SEM image of silver‐coated gold nanoparticles (Au@Ag_PNCs_) enriched on CA filter paper (F_CA_‐Au@Ag_PNCs_ layer), with a red box indicating a magnified section. (D) SEM image of PS microplastics filtered onto the F_CA_‐Au@Ag_PNCs_ layer (F‐F_CA_‐Au@Ag_PNCs_), with a red box indicating a magnified section. (E) SEM image showing redistributed silver‐coated gold nanoparticles uniformly enriched around microplastics. (F) AFM image of CA filter paper. (G) AFM image of Au@Ag_PNCs_ enriched on CA filter paper. (H) FDTD simulated electric field distribution during microplastic filtration on silver‐coated gold nanoparticle‐enhanced CA filter paper. (I) FDTD simulated electric field distribution after refiltration of silver‐coated gold nanoparticles uniformly encapsulating microplastics. (J) Radar chart comparing performance differences among Au@Ag_PNCs_, F_CA_‐Au@Ag_PNCs_, and F‐F_CA_‐Au@Ag_PNCs_ layers in PS microplastic detection, including lowest detectable concentration (LDC), enhancement factor (EF), detection type, signal stability (RSD₁₀₀₀), and field strength (|E/E_0_|). Overall, analyzing the design optimization steps of the reagent kit provides a foundation for its widespread use.

Figure 3A illustrates the design of a microplastic detection platform based on interfacial charge regulation and membrane‐confined hotspot assembly. The system integrates a syringe‐assisted filtration module with a weakly positively charged CA membrane and Au@Ag_PNCs_, enabling controlled nanoparticle assembly and efficient microplastic enrichment.

Guided by the solution‐phase mechanism proposed and supported in Section 2.1, we further engineered the system into a membrane‐confined solid‐phase platform to improve interfacial assembly control and electromagnetic coupling. In this design, negatively charged Au@Ag_PNCs_ are first immobilized onto a weakly positively charged acid‐activated CA membrane to form a dense plasmonic hotspot layer. Subsequent deposition of Au@Ag_PNCs_ results in a vertically stacked bilayer structure that reduces interparticle spacing and promotes interlayer electromagnetic coupling. Thus, the solution‐phase mechanism of charge regulation, interfacial assembly, and plasmonic coupling was translated into a membrane‐confined detection platform, where signal enhancement was governed by local enrichment, hotspot densification, and vertical electromagnetic coupling.

The role of interfacial charge regulation was first supported by zeta potential measurements. After acid activation, the CA membrane surface potential shifted from −1.22 ± 0.21 mV to +2.34 ± 0.28 mV (Figure S12), creating a weakly positive interfacial environment that may facilitate the initial immobilization of negatively charged Au@Ag_PNCs_. However, it is likely that the final nanoparticle loading and assembly density are governed by the combined effects of electrostatic attraction, filtration‐driven deposition, and membrane microstructural confinement [27]. In this design, the weakly positively charged acid‐activated CA membrane serves primarily as an anchoring scaffold for Au@Ag_PNCs_, rather than directly driving microplastic enrichment. In contrast, direct filtration using bare CA membranes led to visible microplastic accumulation near the syringe orifice (Figure 3B), suggesting non‐uniform deposition across the detection area and limited spatial control during filtration. After Au@Ag_PNCs_ were immobilized onto the acid‐activated CA membrane, the resulting Au@Ag_PNC_‐functionalized CA membrane (F_CA_‐Au@Ag_PNCs_) formed a rough, nanoparticle‐rich interfacial layer (Figure 3C). This Au@Ag_PNC_‐based interfacial layer provides abundant plasmonic junctions and increases the apparent hydrophobicity of the membrane surface, as indicated by the increase in contact angle from 49.1° to 124.6° (Figure S13). Enhanced hydrophobicity may enhance interfacial affinity toward hydrophobic microplastic particles, while membrane pores and filtration‐driven transport provide an additional physical enrichment effect. Benefiting from these combined effects, microplastic particles were more uniformly distributed and effectively captured on the substrate (Figure 3D,E), resulting in a high retention rate of 90.54%, calculated from particle number concentrations measured by nanoparticle tracking analysis (NTA) (Text S2). UV–vis spectroscopy was used only as an auxiliary trend analysis tool [28]. SEM and AFM analyses (Figure 3F,G) further reveal a significant increase in surface roughness (Ra from 33.47 nm to 0.254 µm) and nanoparticle packing density, suggesting the formation of more potential hotspot regions. These results demonstrate that charge regulation and wettability modulation synergistically promote localized enrichment and uniform hotspot formation, consistent with the ion‐mediated assembly behavior observed in solution‐phase systems.

To elucidate the interfacial interactions responsible for structural stabilization, XPS chemical‐state analysis was performed (Figure S14). The Ag 3d spectrum can be deconvoluted into two sets of peaks at 367.5/373.6 eV and 368.3/374.3 eV, which are assigned to chemically perturbed Ag species and metallic Ag^0^, respectively (Figure S14D). The chemically perturbed Ag component accounts for 31.4%, suggesting electronic interactions between the Ag surface and the surrounding ligand environment. In addition, the O 1s and S 2p spectra show signals associated with sulfonate groups from PSS‐Na, while the S 2p spectrum can be further fitted into Ag–ligand‐related interfacial species and sulfonate S═O components, accounting for 56.3% and 43.7%, respectively (Figure S14E,F). These results suggest that coordination and/or electrostatic interactions between the sulfonate‐containing PSS‐Na coating and the Ag surface may contribute to stabilizing the assembled nanoparticle layer and improving the reproducibility of local electromagnetic enhancement [29, 30].

To further optimize the structural configuration, the effect of CA membrane pore size on monolayer formation was systematically investigated. Among different pore sizes (5 to 0.1 µm) (Figures S15 and S16), the 0.1 µm membrane produces the most compact and uniform nanolayer (Figure 3C), yielding the highest retention rate (90.54%) and strongest SERS response (Figure S17). This highlights the critical role of microstructural confinement in regulating nanoparticle assembly and hotspot density. While monolayer F_CA_‐Au@Ag_PNCs_ substrates enable efficient enrichment and detectable SERS signals, further enhancement requires increasing the density and connectivity of plasmonic hotspots. Building upon this optimized monolayer, a secondary enrichment and vertical stacking strategy is employed. Additional Au@Ag_PNCs_ were deposited onto the F_CA_‐Au@Ag_PNCs_ substrate through secondary filtration/deposition, which promoted vertical accumulation of Au@Ag_PNCs_ on the preformed plasmonic layer via membrane confinement and filtration‐driven enrichment, resulting in bilayer substrates with increased nanoparticle density and reduced interlayer spacing (F‐F_CA_‐Au@Ag_PNCs_, Figure 3E). In the monolayer system, the characteristic PS peak at 1000 cm^−1^ remained detectable at 10 µg mL^−1^ (S/N > 3, RSD_1000_ = 10.18%, Figure S18A,B). Under identical conditions, the bilayer structure further reduced the lowest detectable concentration to 50 ng mL^−1^ (≈ 5.65 × 10^6^ particles mL^−1^, S/N > 3, Figure S19), representing an improvement of more than two orders of magnitude compared with the monolayer system, with excellent signal reproducibility (RSD_1000_ = 2.57%; inter‐batch RSD = 2.40%, Figure S20). FDTD simulations further revealed that |E/E_0_| increased from 9.62 in the monolayer structure to 17.2 in the bilayer structure, corresponding to an approximately 1.8‐fold enhancement (Figure 3H,I). Experimentally, the SERS enhancement factor increased from 1.34 × 10^4^ to 8.68 × 10^5^ (see Text S3 for detailed EF calculations). It is likely that the larger experimental EF improvement arises from the combined effect of enhanced local electromagnetic fields, increased hotspot density, and improved analyte enrichment, rather than electric field amplification alone.

Taken together, the enhanced SERS performance can be attributed to a sequential and cooperative enhancement mechanism involving weak charge‐assisted nanoparticle immobilization, membrane‐confined microplastic enrichment, hotspot densification, and vertical electromagnetic coupling. The acid‐activated CA membrane facilitates the initial anchoring of negatively charged Au@Ag_PNCs_, while the nanoparticle‐rich rough interface improves local microplastic retention and hotspot accessibility. Secondary Au@Ag_PNCs_ deposition further constructs a bilayer architecture with reduced inter‐particle/inter‐layer spacing, thereby increasing the local electromagnetic field and hotspot density. The observed EF improvement therefore arises from the combined effect of electromagnetic amplification, analyte enrichment, and improved analyte‐hotspot contact, rather than field enhancement alone.

For other types of microplastics (PMMA, PVC, and PC), the system also exhibited good detection capability, with LDCs of 0.5, 1, and 0.5 µg mL^−1^, respectively (S/N > 3, Figure S21). Importantly, stable SERS responses were also obtained at the corresponding LDC levels, with RSD values of 4.57%, 4.72%, and 4.31% for PMMA, PVC, and PC, respectively, based on SERS mapping analysis (Figure S22). These results further demonstrate the reproducibility and cross‐material applicability of the membrane‐confined bilayer SERS platform. Compared with PS (0.05 µg mL^−^^1^), these LDCs were slightly higher, which may originate from differences in intrinsic Raman scattering cross‐sections, particle–substrate affinity, surface chemistry, and the degree of contact with plasmonic hotspots. These results demonstrate the potential versatility and cross‐material adaptability of bilayer structures for the detection of different types of microplastics. Furthermore, comparison with reported microplastic concentrations in aquatic environments (Table S1) indicates that the lowest detectable concentration (LDC) for PS (50 ng·mL^−1^) is comparable to reported levels in certain contaminated aquatic environments. Although the LDCs for PMMA, PVC, and PC are slightly higher, they remain comparable, suggesting the potential applicability of the proposed method for real water sample analysis, while further optimization may be required for complex matrices. In addition, the system exhibits good stability under batch‐to‐batch preparation (RSD = 4.75%), storage stability (RSD = 3.69%), and reusability (RSD = 3.93%), further demonstrating its reliability for practical applications (see detailed analysis in Text S4).

**FIGURE 4:**
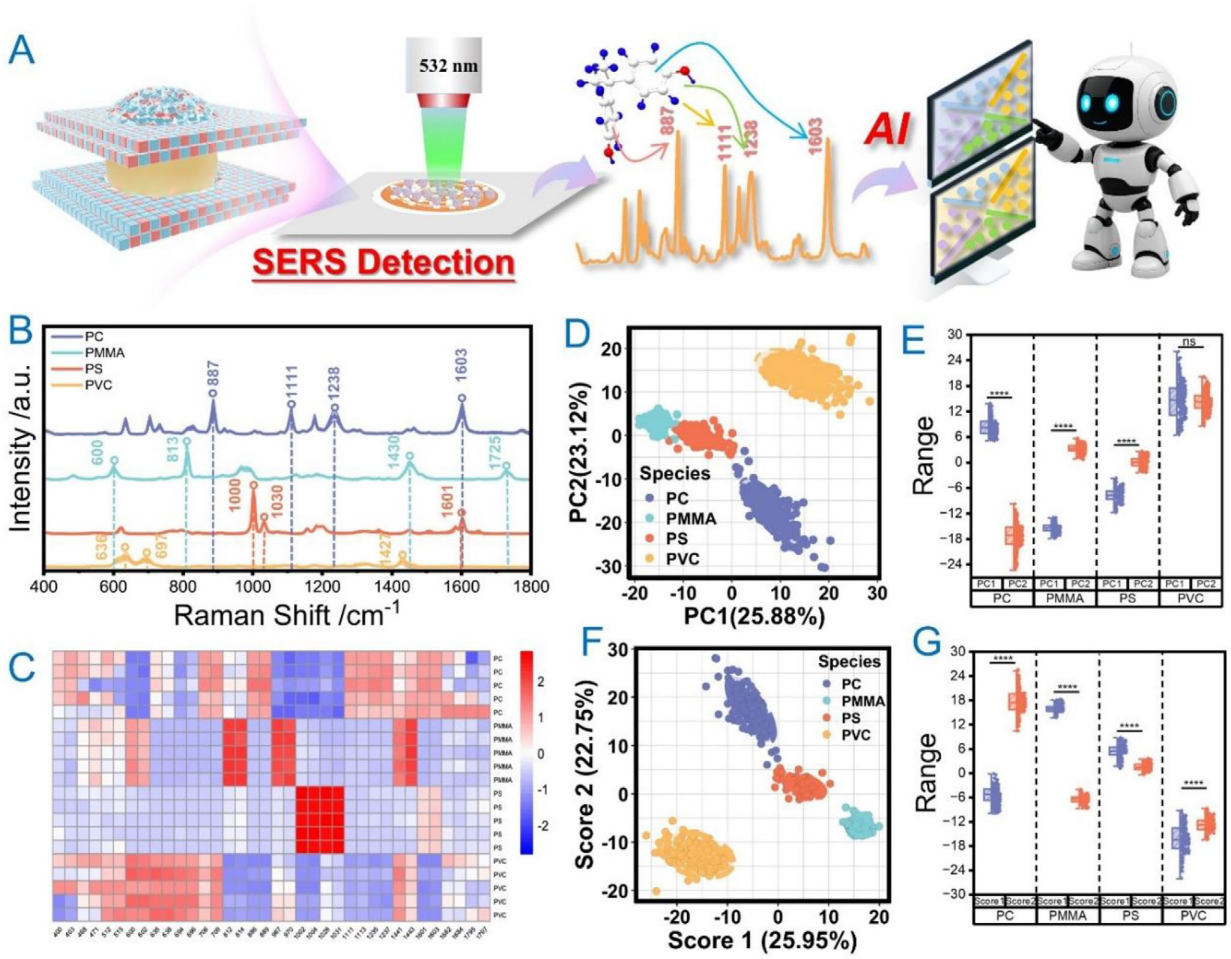
SERS Qualitative identification and machine learning analysis of single‐component microplastic samples. (A) Schematic workflow of single‐component microplastics analysis based on the “dual‐enrichment SERS” strategy. (B) Representative SERS spectra of four microplastics (PC, PMMA, PS, PVC, *n* = 250). (C) SERS thermal maps of four nano/microplastics across the 400–1800 cm^−1^ range. (D) The principal component analysis (PCA) score plot in two dimensions based on SERS spectra. (E) Boxplots of scores for the first two principal components (PC1 and PC2) in PCA. (F) The score plot of partial least squares discriminant analysis in two dimensions (PLS‐DA). (G) Boxplots of the first two PLS‐DA scores (Score1 and Score2), revealing significant differences among different microplastics (^*^ *p* < 0.05, ^**^ *p* < 0.01, ^***^ *p* < 0.001, ^****^ *p* < 0.0001). 250 SERS spectra were randomly collected from each sample for statistical analysis. Overall, the kit has the ability to identify various microplastics, and the use of both supervised and unsupervised algorithms can enable effective differentiation of microplastics.

To further extend the applicability of the platform, additional experiments were performed on microplastic aging and matrix interference. The results indicate that the method remains robust under environmental aging and common dissolved matrix interference, demonstrating its potential applicability in complex environmental samples (see detailed analysis in Text S5). Finally, radar charts (Figure 3J) were employed to compare the performance of three systems (Au@Ag_PNCs_, F_CA_‐Au@Ag_PNCs_, and F‐F_CA_‐Au@Ag_PNCs_) in terms of lowest detectable concentration, enhancement factor (EF), detection capability, signal stability (RSD_1000_), and electric field strength (|E/E_0_|). The corresponding data are presented in Figure S18C and Figure 4B. The results demonstrate that integrating nanoscale interfacial regulation with microscale membrane confinement significantly improves sensitivity, signal stability, and field enhancement. Further comparison with previously reported methods demonstrates that the proposed strategy exhibits significant advantages in sensitivity, multi‐component detection capability, and applicability in complex environments (Table S2).

### 2.3 Qualitative Analysis of Single‐Component Samples

To validate the detection performance of the “dual‐enrichment SERS” assay kit for identifying typical nano/microplastics in aqueous systems, four commonly encountered microplastics in the environment, including PS, PMMA, polyvinyl chloride (PVC), and polycarbonate (PC), were chosen as model analytes (Figure 4A). These represent typical structural units such as aromatic, ester, halogenated, and carbonate groups and cover the chemical diversity of the major microplastics found in the environment. As shown in Figure 4B, all four microplastics exhibit distinct, reproducible SERS fingerprint peaks, demonstrating the excellent signal‐to‐noise ratio and molecular specificity of the system. Among them, PS shows characteristic benzene ring vibration peaks at 1000, 1030, and 1601 cm^−1^ [31]. PVC exhibits C─Cl stretching vibrations at 636 and 697 cm^−1^, while 1430 cm^−1^ corresponds to CH_2_ bending vibrations [32]. PC features characteristic O─C(O)─O and C─O─C vibrations at 887, 1111, and 1238 cm^−1^ [33]. PMMA is associated with methylene CH_2_ stretching and bending vibrations at 813 and 1430 cm^−1^ [34], and the remaining peak assignments are listed in Table S3. To systematically analyze spectral differences among various microplastics, a total of 1000 SERS spectra (250 per type) were collected across the 400–1800 cm^−1^ range for machine learning modeling. Thermal maps (Figure 4C) show that, while overall spectral profiles are similar, identifiable differences exist in local peak positions and signal intensities across samples. Principal component analysis (PCA) was first used to evaluate the intrinsic spectral differences among the four types of microplastics without class‐label supervision (Figure 4D). Although PC1 and PC2 together account for 49.0% of the total variance, the score plots still show distinguishable clustering trends across the four sample types, suggesting that the SERS spectra contain useful molecular fingerprint information for preliminary unsupervised differentiation. Further analysis (Figure 4E) suggests that PC1 contributes mainly to the separation of PS and PC, whereas PC2 is more sensitive to the differentiation of PMMA and PVC, reflecting differences in their characteristic functional groups. This demonstrates that the characteristic components within the SERS spectrum can map differences in molecular functional groups. Building upon this foundation, to further improve identification accuracy, a supervised partial least squares discriminant analysis (PLS‐DA) model was introduced (Figure 4F). The data were split into a training set (700 groups) and a test set (300 groups) at a 7:3 ratio, and a 0.95 confidence ellipse was plotted. The 95% confidence ellipses show compact intra‐class distributions and clear inter‐class separation. The first two scores (Score1 and Score2) collectively explain 48.7% of the variance, with the four microplastic categories forming distinct and without overlapping clustering regions in the space of two dimensions. Further evaluation of model performance via 5‐fold cross‐validation (Figure S23) indicates no overfitting, with an average accuracy of 0.9243 and a standard deviation of 0.0305. Its 95% confidence interval ranges only ±0.0611, demonstrating high model stability and stable classification performance for single‐component microplastic samplesy. Box plot analysis (Figure 4G) showed complete separation of PMMA samples along the Score1 axis, while PS, PC, and PVC showed significant differences along the Score2 axis (^****^ *p* < 0.0001), confirming the PLS‐DA model's high sensitivity discriminative ability. Overall, this “dual‐enrichment SERS” system achieves automated analysis from “signal acquisition” to “intelligent discrimination” through spectral amplification of structural differences and algorithmic recognition. Molecular structural features within the SERS signal are quantified and mapped using a machine learning model, enabling rapid qualitative identification of nano/microplastics. Compared with traditional manual peak identification methods, this approach establishes an integrated “structure–spectrum–algorithm” detection pathway, improving the accuracy and automation of microplastic identification and providing a basis for future analysis of complex environmental samples.

### 2.4 Qualitative Analysis of Multiple Component Samples

**FIGURE 5:**
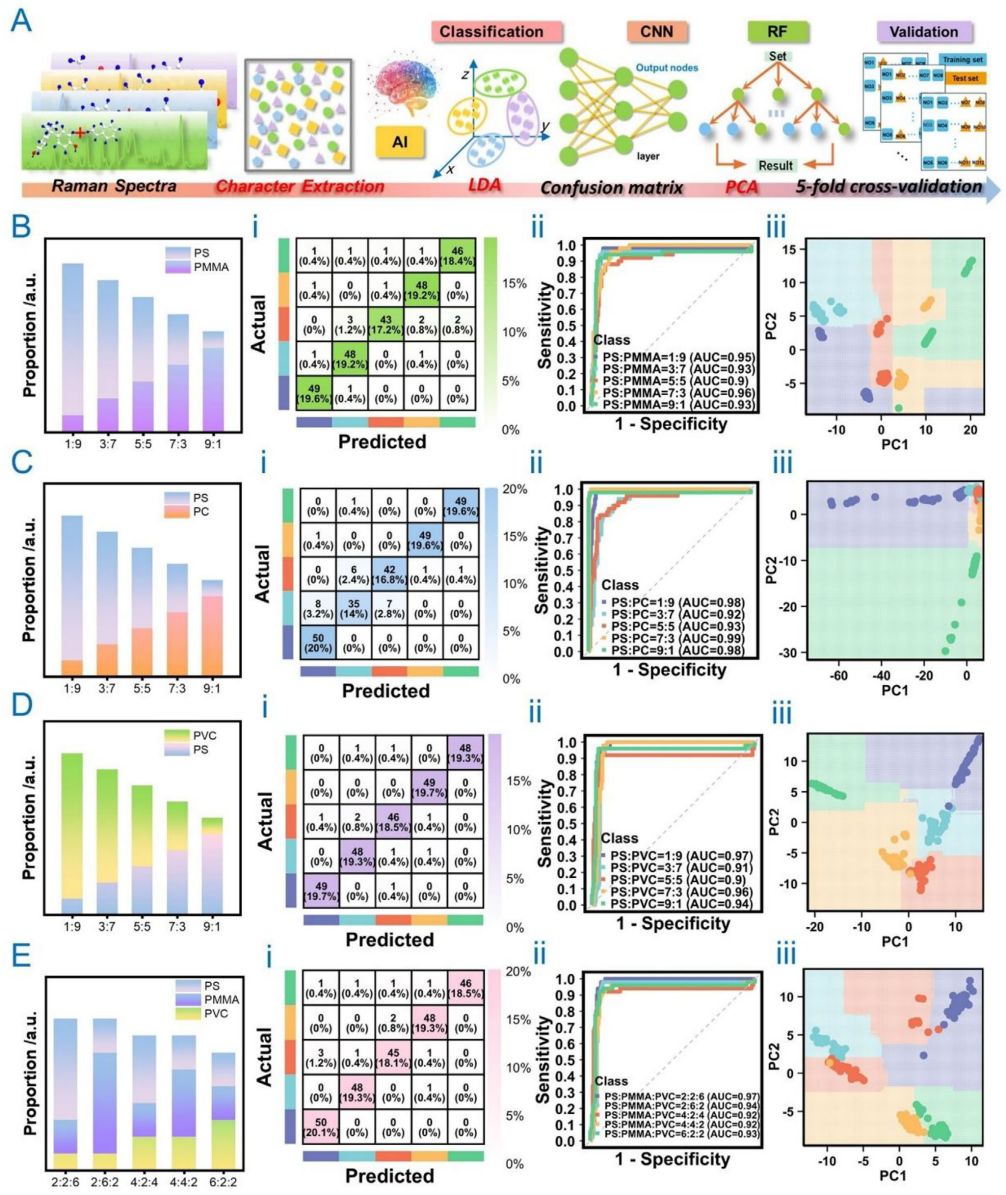
Qualitative SERS identification and machine learning classification of multiple component microplastic samples. (A) Schematic of the machine learning framework for classifying and identifying multiple component microplastics using SERS data. (B–D) Qualitative identification results of binary mixtures (PS: PMMA, PS: PC, PVC: PS), including confusion matrices (i), ROC curves (ii), and CNN‐RF decision boundaries (PCA 2D visualization) (iii). 250 SERS spectra were randomly collected for each sample ratio (1:9, 3:7, 5:5, 7:3, 9:1). (E) Recognition results for ternary blends (PS: PMMA: PVC) at different ratios, including a confusion matrix (i), ROC curve (ii), and CNN‐RF decision boundary (iii) (*n* = 250). Overall, the results validate the high stability and generalization capability of the CNN‐RF model in multiple component systems.

Nanoplastic and microplastic contamination in real water bodies often coexist as a mixture of multiple components. The chemical composition and Raman properties of different polymers are very similar, especially for microplastics with C═C or C─Cl bonds. Their SERS signals tend to overlap and interfere with each other, making qualitative identification much more difficult [35]. Therefore, reliable identification of hybrid microplastic systems requires not only spectral enhancement but also data‐driven feature extraction and classification. This study developed a machine learning framework that integrates classification, prediction, evaluation, and cross‐validation (Figure 5A; Figure S24) to enable intelligent identification of multicomponent microplastics in complex systems. In terms of model design, linear discriminant analysis in three dimensions (3D‐LDA) was initially used for supervised dimensionality reduction and classification of SERS data of high dimensions, highlighting the clustering patterns of different components within the feature space. Next, a convolutional neural network (CNN) was implemented as a spectral feature extractor, automatically extracting discriminative spectral features. These deep features were then fed into a random forest (RF) classifier for discrimination. This “CNN‐RF” hybrid model combines the feature abstraction strengths of deep learning with the robustness of ensemble learning. Model performance is evaluated using ROC curves and AUC values, and stability and reliability are confirmed by 5‐fold cross‐validation. In the two‐component systems (PS: PMMA, PS: PC, PS: PVC), five ratios were used: 1:9, 3:7, 5:5, 7:3, and 9:1 (for the detailed experimental procedure, see Text S6), with 1250 spectra collected for each group. The 3D‐LDA results show clear clustering and separation among different binary mixture ratios in the projected feature space (Figure S25A–C). The first three discriminative components contribute dominantly to class separation, indicating that the SERS spectrum contains sufficient compositional information for supervised differentiation of multi‐component systems. The AUC values of the ROC curves for all systems exceeded 0.90 (Figure S26), confirming the model's excellent performance in terms of sensitivity and specificity. Further predictions were made using the CNN‐RF model (Figure 5Bi–Di). The CNN‐derived features contributed to effective classification, while the RF achieved high precision classification, with prediction accuracy surpassing 90.00% across all sample proportions. Confusion matrix analysis showed that the three main evaluation metrics, including Precision, Recall, and F1‐score, all exceeded 0.975 (Table S4), indicating the model's strong performance in multiple component systems. The ROC curves displayed AUC values above 0.90 (Figure 5Bii–Dii), indicating robust classification performance and stable discrimination capability under the present experimental conditions. The RF feature importance ranking identified the top 15 features as the most influential, confirming that the CNN‐learned spectral features have sufficient discriminative power (Figure S27). Visualization of decision boundaries revealed sharply defined color regions with evenly distributed test samples (Figure 5Biii‐Diii). Analysis showed nearly identical performance between the training and test sets (ga*p* < 0.1), suggesting no obvious overfitting tendency and stable model behavior under the current dataset and training conditions (Figure S28).

Building on the two‐component results, a ternary blend system (PS, PMMA, PVC) was further developed with five ratio configurations: 2:2:6, 2:6:2, 4:2:4, 4:4:2, and 6:2:2. The 3D‐LDA results show clear separation in the projection space, and the ROC curve AUC values exceeded 0.90 (Figures S25D and S29A). The CNN‐RF model outcomes also reached Accuracy = 0.9518 (Figure 5Ei). Other metrics, including Precision, Recall, F1‐score (Table S4), also reached 0.9879, and the ROC curve AUC all reached 0.92 (Figure 5Eii). Figure 5iii shows that the test samples are uniformly distributed. Analysis of the top 15 feature dimensions (Figure S29B) indicates the model maintains excellent scalability and stability within the ternary system. Lastly, performance analysis of the training and testing sets (Figure S29C) suggests no obvious overfitting tendency and stable classification performance within the tested ternary system.

The robustness of the model was evaluated using 5‐fold cross‐validation (Figures S29D,E and S30). The results show that the average accuracy across all folds is above 0.925 for both binary and ternary systems. Specifically, the average accuracy exceeded 0.923, with a standard deviation below 0.0196 and a 95% fluctuation range below 0.0391 (Table S5), demonstrating the reproducibility and reliability of the model across different sample subsets. In summary, the CNN‐RF assisted SERS analysis framework improves the qualitative discrimination of binary and ternary microplastic mixtures under controlled experimental conditions. Compared to conventional spike‐based SERS analysis, this strategy enables more reliable multi‐component classification by integrating spectral enhancement with data‐driven feature extraction. These results provide a proof‐of‐concept basis for extending machine‐learning‐assisted SERS analysis toward more complex environmental microplastic systems in future studies.

### 2.5 Database Establishment and Similarity Analysis

**FIGURE 6:**
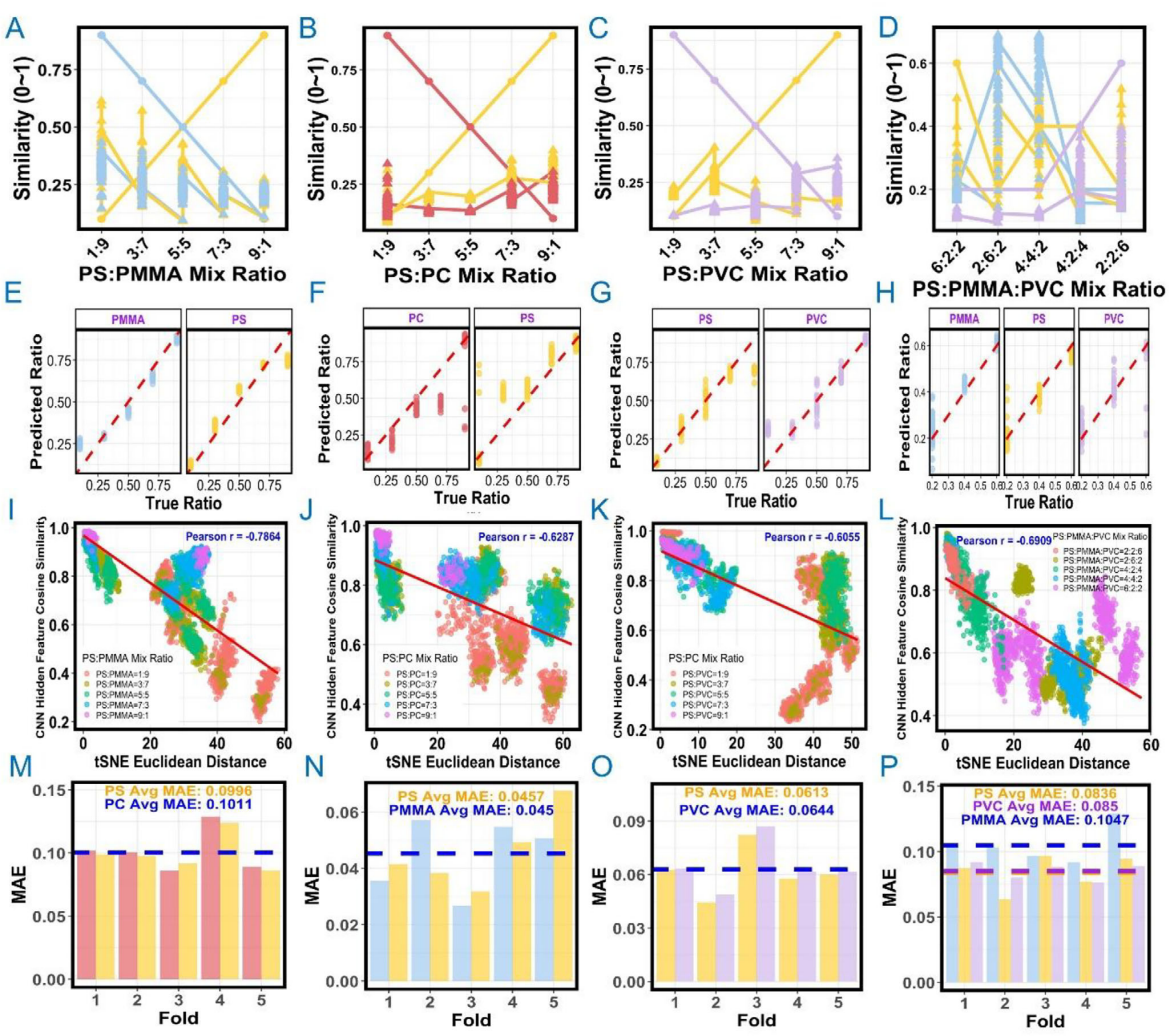
Construction and application of the similarity analysis database. (A–D) Similarity calculations of SERS spectra from different systems using Euclidean distance, PS: PMMA (PS, light yellow, PMMA, light blue), PS: PC (PC, light red, PS, light yellow), PS: PVC (PVC, light purple, PS, light yellow), and ternary system PS: PMMA: PVC (PMMA, light blue, PVC, light purple, PS, light yellow). Dots represent theoretical ratios, while triangles denote calculated similarity. (E–H) Comparison of actual versus predicted proportion based on the 1D‐CNN model, where points closer to the diagonal indicate higher similarity. (I–L) Pearson correlation analysis between cosine similarity of CNN model hidden layer features and t‐SNE distances, showing distinct clustering of samples with different ratios in the space of two dimensions. (M–P) Mean Absolute Error (MAE) results under 5‐fold cross‐validation for each system. Overall, demonstrating high stability and generalization performance of the model in multiple component systems.

Although classification models have greatly improved the accuracy of microplastic identification, reusable experimental SERS spectroscopic databases for rapid identification and semi‐quantitative analysis of unknown samples are still limited. Because different microplastics share chemical similarities and overlapping SERS spectral features, traditional similarity‐based algorithms often struggle to achieve high‐precision differentiation. To address this issue, this study builds a discriminative database that combines traditional similarity computation with deep feature learning. This allows for adaptive extraction of spectral features and prediction of component ratios, creating a data foundation for intelligent microplastic identification and compositional estimation [36, 37, 38].The database includes four common microplastics (PS, PMMA, PC, PVC). All single‐component spectra were normalized using Min–Max normalization and screened with PCA to select high‐variance dimensions, thereby optimizing the CNN input feature space (250 sets per category) (Figure S31). Traditional similarity analysis was first performed using Euclidean distance and cosine similarity. Taking the PS: PMMA system (ratios 1:9, 3:7, 5:5, 7:3, 9:1) as an example, results showed (Figure 6A) that the mean absolute error (MAE) for PS and PMMA was 0.343 and 0.294, respectively, indicating low matching accuracy. After optimizing parameters, cosine similarity was recalculated, but results showed no significant improvement (Table S6). A similar trend was observed for PS: PC, PS: PVC, and the ternary system PS: PMMA: PVC (Figure 6B–D; Table S7), indicating that conventional distance algorithms based on geometry struggle to capture the nonlinear spectral differences caused by chemical bonding and plasmon resonance coupling in SERS spectra. To address this limitation, a one‐dimensional convolutional neural network (1D‐CNN) combined with a channel attention mechanism was introduced. By adaptively assigning weights to focus on regions with chemical features, the model learns the nonlinear mapping between spectral data and compositional ratios. This approach enables “adaptive similarity” calculations, greatly improving discrimination and predictive performance. In the PS: PMMA system (Figure 6E), the 1D‐CNN model achieved a mean absolute error (MAE) of 0.0479 for PS with R^2^ > 0.96, and an MAE of 0.0494 for PMMA with R^2^ > 0.96. The predicted ratio points are clustered near the theoretical diagonal, accurately reflecting the ratio relation. For PS: PC, PS: PVC, and the ternary system (Figure 6F–H), the MAE remained below 10%, and the coefficient of determination (R^2^) exceeded 0.83 (Tables S8 and S9), demonstrating the model's applicability across the tested binary and ternary systems. The training and validation loss curves (Figure S32) converged quickly and stabilized within 50 epochs, suggesting stable convergence and no obvious overfitting tendency under the present training conditions. Predicted proportion deviations clustered near zero (Figure S33), ensuring proportion conservation in validation. t‐SNE dimensionality reduction analysis (Figure 6I–L) showed clear separation of samples in the space of two dimensions, with similar samples forming good clusters. Moreover, the negative correlation between t‐SNE distances and CNN feature cosine similarity (Pearson < −0.60) suggested that the learned latent features may retain compositional relevance rather than merely providing class separation. 5‐fold cross‐validation results (Figure 6M–P) showed MAE consistently below 0.105, further confirming the model's stability and robustness in multiple component systems. In summary, the similarity analysis database based on 1D‐CNN and attention mechanisms shifts from “geometric distance similarity” to “feature learning similarity.” This improves the semi‐quantitative prediction capability of multi‐component microplastic systems, providing an efficient method for fast identification and proportion estimation of unknown samples. It should be emphasized that the model provides semi‐quantitative compositional estimates rather than strict absolute quantification. While the predicted relative polymer fractions agree well with the nominal ratios under controlled conditions, further calibration with environmentally aged particles, complex matrices, and concentration‐dependent response factors is still required before a rigorous quantitative application to real environmental samples.

### 2.6 Authentic Sample Analysis

**FIGURE 7:**
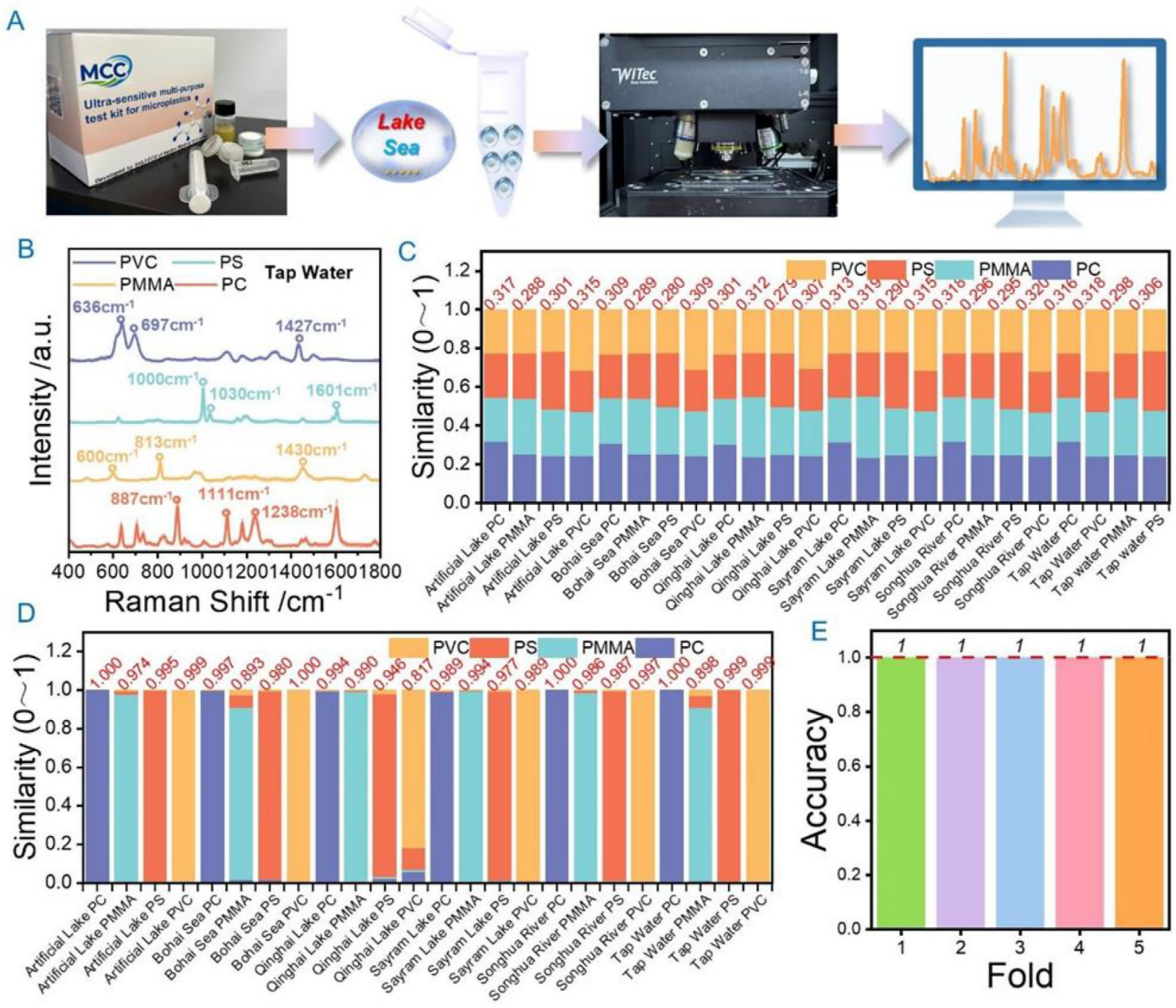
SERS Detection and similarity identification of many type microplastics in real water systems. (A) Schematic of the overall workflow for detecting microplastics in complex water systems using the "dual‐enrichment SERS" detection kit. (B) SERS characteristic peak intensity plots for four typical microplastics, including PVC, PS, PMMA, and PC in tap water, *n* = 50). (C) Results of spectral similarity matching using Euclidean Distance (ED). (D) Similarity recognition results for four types of microplastics in six water bodies using a convolutional neural network (CNN) model (red numbers indicate similarity values for corresponding components). (E) 5‐fold cross‐validation results for the CNN model, demonstrating high stability and reproducibility. Overall, the excellent similarity recognition results fully demonstrate the feasibility of the designed similarity model and provide a general paradigm for environmental monitoring.

To validate the effectiveness and stability of the constructed SERS detection kit in complex aquatic environments, six representative environmental water matrices including tap water, river water, lake water, rain water, groundwater, and seawater were selected for real‐world sample validation. The four common microplastics, PS, PMMA, PC, and PVC, were separately spiked into each water matrix at a final concentration of 1mg mL^−^^1^. Variations in pH, salinity, and organic matter in ambient water often reduce the sensitivity of traditional detection methods and lead to poor signal reproducibility. Therefore, it is crucial to evaluate the system resistance to interference under real‐world conditions. Figure 7A shows the detection workflow, including kit assembly, standard addition sample preparation, SERS signal collection, and database comparison, forming a complete detection cycle from sample collection to identification. The resulting SERS signals consistently exhibit high resolution and strong signal‐to‐noise across a variety of aquatic environments, demonstrating robust detection capabilities. Taking tap water as a representative example (Figure 7B), the characteristic SERS peaks of the four microplastics remained clearly distinguishable. In particular, polyvinyl chloride (PVC) exhibited characteristic peaks at 636 and 697 cm^−^^1^, corresponding to C─Cl stretching vibrations, while polystyrene (PS), polymethyl methacrylate (PMMA), and polycarbonate (PC) also retained their characteristic spectral fingerprints without obvious peak shifts or severe signal distortion. Similar spectral behavior was observed in the other five water matrices (Figure S34), where the characteristic peaks of all four microplastics remained identifiable under different environmental conditions. The results indicate that this detection method exhibits excellent anti‐interference stability against matrix variations of actual water environments. A real sample database was onstructed using SERS spectra collected from diverse environmental systems for feature comparison and model training of multi‐component microplastics. When applying traditional Euclidean distance for spectral matching, similarity scores were generally low (maximum 0.319) (Figure 7C), indicating limited capability in resolving nonlinear spectral differences in complex systems. Therefore, a convolutional neural network (CNN) was introduced for deep feature learning and classification. The CNN extracts key spectral features through convolutional feature extraction and nonlinear mapping, thereby improving spectral discrimination and noise resistance. Results (Figure 7D) show recognition accuracies above 0.82 for all water samples and microplastics, with some systems reaching 1.0.5‐fold cross‐validation showed relatively small variations among folds, with consistently high classification accuracy (Figure 7E), suggesting stable model performance under the tested conditions. Notably, these results were achieved without acid digestion, oxidative purification, or complex separation steps [39, 40, 41]. Identification is done using only a simple process of filtration, enrichment, SERS collection, and model discrimination, demonstrating high efficiency and suitability for field deployment under minimal pretreatment conditions. In summary, the constructed “dual‐enrichment SERS” detection kit, combined with a deep‐learning‐assisted recognition model, enables rapid and stable identification of multiple types of microplastics in standard‐spiked real water samples. These results demonstrate the potential of the platform for future on‐site microplastic screening and environmental monitoring, although further validation using naturally contaminated samples and more complex environmental matrices is still required.

## 3 Conclusion

In summary, this work develops a membrane‐confined SERS platform integrated with machine‐learning‐assisted spectral analysis for sensitive and reproducible microplastic detection in complex aquatic environments. The enhancement process is governed by the cooperative effects of membrane‐guided microplastic enrichment, stable nanoparticle‐microplastic contacts, hotspot densification, and vertical electromagnetic coupling, resulting in a dense and reproducible network of electromagnetic hotspots. As a result, the platform achieves a lowest detectable concentration of 50 ng mL^−1^ for polystyrene and demonstrates good signal reproducibility in microplastic spike samples prepared in six environmental water matrices without chemical pretreatment. To address the spectral similarity among polymers, a multi‐polymer SERS spectral library is constructed and combined with an attention‐based 1D convolutional neural network. This machine‐learning‐assisted approach enables accurate identification of four common microplastics and semi‐quantitative analysis of binary and ternary mixtures. Representative polymers, including PS, PMMA, PVC, and PC, exhibit stable and reproducible SERS responses, indicating good applicability across materials. However, the SERS response of other microplastics may vary depending on their intrinsic Raman activity, surface properties, and interactions with nanostructures. Therefore, further validation in more complex environmental systems is required. Overall, the integration of membrane‐confined microplastic enrichment, bilayer hotspot construction, and data‐driven spectral identification provides a robust strategy for sensitive and reliable microplastic detection in environmental water systems.

## 4 Experimental Section

### 4.1 Materials

Silver nitrate (AgNO_3_) was obtained from Alfa Aesar (China). Sodium borohydride (NaBH_4_) came from Sigma–Aldrich (USA). CTAB, CTAC, PVC, and PSS‐Na were supplied by Macklin (Shanghai). L‐Ascorbic acid was purchased from Sangon Biotech (Shanghai). Gold (III) chloride trihydrate (HAuCl_4_·3H_2_O) was provided by Xiya Chemical (Shandong). Polystyrene microspheres were sourced from Aladdin (Shanghai). Acetate filter paper and 25 mm membrane filters were obtained from Haining Deflu, and 10 mL syringes from Jiangxi Hongda. Tap water was collected in Harbin, while lake, river, seawater, Qinghai Lake water, and Sayram Lake water were sampled from their respective locations. A saltwater hydrometer was purchased from Taobao (China).

### 4.2 Preparation of the Gold Nanoparticle Seed

Under stirring conditions, a 0.2 mL, 0.01 m solution of HAuCl_4_·3H_2_O was added to a 8 mL solution of CTAB (0.1 m). Stirring was continued, then 0.6 mL of freshly prepared cold NaBH_4_(0.01 m) solution was added quickly, stirred for 3 min, and left to settle for more than 1 h and set aside.

### 4.3 Preparation of Gold Nanocubes

The 0.0583 g CTAB was dissolved in 9.6 mL of H_2_O by sonication. Then 0.2 mL of 0.01 m HAuCl_4_·3H_2_O solution was added, and the solution changed from colorless to bright yellow. Under the condition of stirring, 0.9 mL of AA solution (0.1 m) was added, the color changed from bright to colorless yellow. Take 5 µL of tenfold diluted seed solution and add it to the growth solution, react at 50°C for 15 min, set aside.

### 4.4 Preparation of Silver Coated Gold Nanocubes

Silver‐coated gold nanocubes solution was centrifuged at 7000 rpm·min^−1^, washed 2 times and redispersed into 10 mL of CTAC solution (20 mm). Then 50 µL of AgNO_3_ solution (0.1 m) was added and stirred at 60°C for 20 min. Finally 50 µL of AA solution (0.5 m) was added and stirred for 4 h to obtain silver coated gold nanoparticles. Silver‐coated gold nanoparticles were centrifuged for 15 min at 7000 rpm·min^−1^ and washed three times. The ligand exchange reaction is then performed by adding a certain amount of 0.5% to 2% PSS‐Na anionic surfactant, which results in a modified cubic colloid.

### 4.5 Preparation and Detection Procedures for the Kit

First, a weakly positively charged CA membrane (treated with dilute HCl) was assembled with the filtration device. Subsequently, 100 mL of synthesized Au@Ag_PNCs_ nanocubes was collected by centrifugation, washed, surface modified with PSS‐Na, and concentrated to 10 mL. A 5 mL concentrated nanoparticle solution was then introduced onto the membrane using a syringe under progressive pressure to achieve uniform enrichment of the membrane surface. This process was repeated 4–5 times until a homogeneous nanostructured layer was formed. Next, the target microplastic sample was filtered and immobilized onto the first nanoparticle‐enriched membrane layer. An additional 5 mL of concentrated nanoparticle solution was subsequently applied under progressive pressure, enabling further enrichment around the microplastics and the formation of a coating structure, thereby generating an enhanced SERS signal.

### 4.6 SERS Detection

Raman measurements were performed using a Raman AFM system (WITec 𝛼300 RA, Germany) with the following parameters, laser wavelength: 532 nm, grating: 600 grooves min^−1^, resolution: 3 cm^−1^, scanning time: 10 s, and laser power: 15 mW, and 1 accumulation. All measurements were conducted under identical conditions unless otherwise specified.

### 4.7 XPS Analysis

The membrane samples were cut into pieces of approximately 5 × 5 mm and mounted onto the sample holder. X‐ray photoelectron spectroscopy (XPS) measurements were carried out using a Thermo Scientific K‐Alpha spectrometer. The samples were first introduced into the loading chamber, and subsequently transferred to the analysis chamber when the pressure was below 2.0 × 10^−^^7^ mbar. The x‐ray spot size was 400 µm, with an operating voltage of 12 kV and a filament current of 6 mA. Survey spectra were recorded with a pass energy of 150 eV and a step size of 1 eV, while high‐resolution spectra were collected with a pass energy of 50 eV and a step size of 0.1 eV. Spectral analysis was performed using Avantage software (Thermo Fisher Scientific). To accurately determine the chemical state of the elements, all binding energies were calibrated using the C1s peak at 284.8 eV as a reference.

### 4.8 Statistical Analysis

All statistical analyses were performed using GraphPad Prism 8.0 software. Data preprocessing included outlier detection and normality assessment. The SERS spectra were collected from independent replica measurements performed under the same experimental conditions. For the analysis of binary and ternary mixtures, 250 independent SERS spectra were collected for each component, while the sample sizes (n) for the remaining experiments are clearly specified in the corresponding figure legends and in the relevant sections of the paper. Box plot data are presented as median and interquartile range (IQR). Statistical comparisons were performed using an unpaired two‐tailed Student's t‐test where appropriate. The significance threshold was set at α = 0.05, and statistical significance was defined as follows: ^*^ *p* < 0.05; ^**^ *p* < 0.01; ^***^ *p* < 0.001; and ^****^ *p* < 0.0001.

## Author Contributions

The manuscript was written through contributions of all authors. All authors have given approval to the final version of the manuscript.

## Conflicts of Interest

The authors declare no conflicts of interest.

## Supporting information




**Supporting File**: smll74231‐sup‐0001‐SuppMat.docx.

## Data Availability

Author elects to not share data.
