# Supplementary material for: Interfacial Regulation–Driven Dual‐Enrichment SERS Coupled With Deep Learning Enable Ultrasensitive and In Situ Identification of Microplastics in Natural Waters
Source: Small. 2026 Jun 19;22(44):e74231. doi: 10.1002/smll.74231 (PMC13450292; doi:10.1002/smll.74231)
Supplement: Supplementary file 1 [file SMLL-22-e74231-s001.docx]

Supporting Information

**Interfacial Regulation–Driven Dual-Enrichment SERS Coupled with Deep Learning Enable Ultrasensitive and In Situ Identification of Microplastics in Natural Waters**

*Chaochao Ma^a^#, Xiaojiao Zhao^a^#, Jiacheng Li^a^, Xinyu Liu^a^, Mingxu Zhang^a^, Zhidan Liu^a^, Jing Wu^e^, Zhifeng Liu^c^*, Yunpeng Wang^a^*, and Yang Li^a,b,c,d^**

*^a^ State Key Laboratory of Frigid Zone Cardiovascular Diseases (SKLFZCD), Research Center for Innovative Technology of Pharmaceutical Analysis, College of Pharmacy, Harbin Medical University, Harbin City, Heilongjiang Province, 150081, China.*

*^b^ Research Unit of Health Sciences and Technology (HST), Faculty of Medicine, University of Oulu, Oulu, 90014, Finland.*

*^c^ Inpatient Department, Heilongjiang Provincial Eye Hospital, Harbin City, Heilongjiang Province, 150081, China.*

*^d^ Heilongjiang provincial Clinical Medical Research Center for Chemical Poisoning, Heilongjiang second provincial Hospital, Harbin City, Heilongjiang Province, 150081, China.*

*^e^ School of Physical Science and Technology, Nantong University, Nantong, Jiangsu Province, 226019, China.*

\# Chaochao Ma and Xiaojiao Zhao contributed equally to this work.

* Corresponding author Email:

Yunpeng Wang Email: wyp0529@126.com.

Zhifeng Liu Email: lzf1972@163.com.

Yang Li Email: Yang.Li@oulu.fi; liy@hrbmu.edu.cn.

**Table of Contents**

1. **Machine Learning:** PLS-DA, PCA, 3D-LDA, CNN-RF, Database Construction, Similarity Analysis and Synthesis of PC and PVC microplastics
2. **Figure S1** SEM image of Au@Ag_NCs_.
3. **Figure S2** Elemental mapping of Au@Ag_NCs_.
4. **Figure S3** High resolution TEM image of Au@Ag_NCs_.
5. **Figure S4** XRD pattern of Au@Ag_NCs_ nanoparticles.
6. **Figure S5** UV vis absorption spectra of Au_NCs_ and Au@Ag_NCs_.
7. **Figure S6** SERS spectra of PS microplastics at various concentrations using Au@Ag_NCs_.
8. **Figure S7** Stability evaluation of Au@Ag_NCs_ detection signals.
9. **Figure S8** TEM image of Au@Ag_NCs_ adsorbed onto PS microplastic surfaces.
10. **Figure S9** Raman spectra before and after modification.
11. **Figure S10** SERS spectra of PS microplastics using Au@Ag_PNCs_ with different aggregation-inducing ions (Zn²⁺, Na⁺, Mg²⁺, Ca²⁺).
12. **Figure S11** Average particle size distribution of PS microplastics and Au@Ag_PNCs_ before and after Al^3+^ addition.
13. **Figure S12** Zeta potential of CA filter paper before and after modification with 1 mL of 10 mM dilute hydrochloric acid solution.
14. **Figure S13** Contact angle images of CA filter paper.
15. **Figure S14** XPS survey of cellulose acetate filter paper (CA), F_CA_-Au@Ag_NCs_ and F_CA_-Au@Ag_PNCs_.
16. **Figure S15** SEM image of CA filter paper(0.1μm).
17. **Figure S16** Scanning electron microscopy (SEM) images of CA filter paper with different pore sizes.
18. **Figure S17** SERS spectra of PS microplastics enriched on CA Filter paper with different pore sizes using Au@Ag_PNCs_.
19. **Figure S18** Evaluation of the stability and sensitivity of F_CA_-Au@Ag_PNCs_ layer detection.
20. **Figure S19** SERS spectra of PS microplastics at various concentrations on the F-F_CA_-Au@Ag_PNCs_ layer.
21. **Figure S20** Raman mapping of PS microplastics on the F-F_CA_-Au@Ag_PNCs_ layer and the stability of PS microplastic detection.
22. **Figure S21** SERS spectra of PMMA, PVC and PC at different concentrations.
23. **Figure S22** SERS mapping images of different microplastics on the F-F_CA_-Au@Ag_PNCs_ substrates at their corresponding LDC levels.
24. **Figure S23** Bar plot of 5-fold cross-validation results for the PLS-DA model.
25. **Figure S24** SERS spectra for PS/PMMA, PS/PC, PS/PVC, and PS/PMMA/PVC mixtures.
26. **Figure S25** 3D-LDA score plots of binary/ternary mixtures.
27. **Figure S26** ROC curves and corresponding AUC values for 3D-LDA classification in multicomponent binary systems.
28. **Figure S27** Multi component systems top 15 feature dimension plot.
29. **Figure S28** Overfitting validation of CNN models for multi-component microplastic systems.
30. **Figure S29** The ROC plots of the PS : PMMA : PVC systems and 5-fold cross-validation and overfitting verification of CNN models.
31. **Figure S30** Bar plots of fold-wise accuracy from 5-fold cross-validation of the CNN model for binary microplastic systems.
32. **Figure S31** Schematic diagram of the overall computational workflow for establishing the spectral database and performing similarity analysis.
33. **Figure S32** Trends in various metrics of CNN models during training for multiple component systems (PS: PMMA, PS: PC, PVC:PS, PS: PMMA: PVC).
34. **Figure S33** Box plots of prediction deviation for different system ratios using the 1D-CNN model.
35. **Figure S34** SERS characteristic peak intensity plots for four typical microplastics in different water systems.
36. **Table S1** The detection limit of this study compared with that of the reagent in the aquatic environment.
37. **Table S2** Comparison of our method with the conventional SERS method for microplastics detection.
38. **Table S3** SERS peak assignments for microplastic.
39. **Table S4** Classification performance metrics of CNN models for binary and ternary microplastic mixtures.
40. **Table S5** 5-fold cross-validation performance metrics for binary and ternary microplastic mixtures.
41. **Table S6** MAE values of binary microplastic mixtures from Euclidean Distance and cosine similarity analysis.
42. **Table S7** MAE values of ternary microplastic mixtures from Euclidean Distance and cosine similarity analysis.
43. **Table S8** Adaptive Learning Improves MAE and R² in Binary Polymer CNN Models.
44. **Table S9** Adaptive Learning Improves MAE and R² in Ternary Polymer CNN Models.
45. **Text S1** Additional characterization.
46. **Text S2** Determination of membrane retention efficiency for PS microplastics using nanoparticle tracking analysis.
47. **Text S3** Calculation of the Enhancement Factor for PS.
48. **Text S4** Evaluation of reproducibility and stability.
49. **Text S5** Microplastic aging and matrix interference assessment.
50. **Text S6** Qualitative analysis and data processing workflow.

**Machine Learning**

**PLS-DA:** The Partial Least Squares – Discriminant Analysis (PLS-DA) algorithm was implemented using R 4.4.2, calling the “plsda” function in the mixOmics package and the ggplot2 package. PLS-DA algorithm. The “ggplot” function is used to plot the PLS-DA graph, where Score1 is mapped to the horizontal axis and Score2 is mapped to the vertical axis, and the “stat_ellipse” function is used to plot the ellipse of 95% confidence error.

**PCA:** Principal component analysis (PCA) was performed using R 4.4.2 by calling the prcomp function of the stats package and the ggplot2 package. The “ggplot” function was used to plot the PCA plot, where PC1 was mapped to the horizontal axis and PC2 to the vertical axis, and the “stat_ellipse” function was used to plot the 95% confidence error ellipse.

**3D-LDA:** Linear Discriminant Analysis (LDA) was performed using R 4.4.2 by calling the lda function of the MASS package, along with the ggplot2, caret, pROC, and plotly packages. Prior to analysis, feature variables were subjected to Min-Max normalization, and the dataset was divided into training and testing sets using the create Data Partition function from the caret package. The lda function was used to train the LDA model, and the explained variance ratios of the linear discriminants (LDs) were calculated. LD scores were obtained using the predict function, and these scores, combined with species labels, were used for visualization. A 3D scatter plot of LDA results was generated using the plotly package, where LD1, LD2, and LD3 (when available) were mapped to the x, y, and z axes.

**CNN-RF:** In R 4.4.2, first, CSV data is imported. After Min-Max normalization, it is stratified into training and testing sets in a 7:3 ratio, and the features are adjusted to the 3D tensor format required by the 1D CNN. A CNN model with convolutional blocks, fully connected layers, and Dropout is built using keras. It is trained through functions such as layer_conv_1d and fit. Then, the high dimensional features extracted by the CNN are input into a random forest using the randomForest function to construct a CNN+RF model. Finally, both models are evaluated using confusion matrices, ROC curves, etc., to obtain results such as test set accuracy and macro average metrics, and their performance differences are compared, and last its stability is evaluated via 5-fold cross validation.

**Database Construction and Similarity Analysis:** In R, first import the database with the "Species" label and the CSV data of the unknown system, aggregate features by group, perform Min-Max normalization, conduct PCA dimensionality reduction using prcomp to construct a database (saving the PCA model, features, and metadata), then calculate the similarity between the unknown group and the database groups using euclidean_dist and cosine_sim. Subsequently, use keras to build a 1D-CNN (including layer_conv_1d convolution blocks, fully connected layers, and Dropout).After training, predict the category of the unknown system, visualize the features of the convolution layers, extract features from the fully connected layers of the CNN to calculate cosine similarity, and use tSNE to visualize the data after dimensionality reduction. Finally, perform 5-fold cross validation via create Folds to evaluate stability.

**Synthesis of PC and PVC microplastics:** First, purchase pure PVC and PC plastic products, cut them into small pieces of approximately 2×2 mm, and completely dissolve 300 mg of the plastic products in 20 mL of organic solvent. Dichloromethane is used to dissolve PVC plastics, while chloroform is used for PC plastics. Add 0.09 g of CTAB to 9 mL of the dissolved plastic solution. After the CTAB is completely dissolved, add 1 mL of water and sonicate for 5 minutes. Next, mix the CTAB aqueous solution (1.75 g, 87.5 mL) with the above emulsion and further emulsify for 4 minutes. To evaporate the organic solvent, the resulting emulsion is then placed in a water bath set at 50°C. After the organic solvent has completely evaporated, centrifuge at 14,000 rpm for 20 minutes to concentrate. Repeat the wash redispersion process 4 times and finally disperse in water to obtain PVC and PC microplastics with a concentration of 10 mg mL^-1[1, 2]^.

**Supplementary Figures**

**Figure S1** SEM image of Au@Ag_NCs_.

**Figure S2** Elemental mapping of Au@Ag_NCs_, confirming the typical core shell structure.

**Figure S3** High resolution TEM image of Au@Ag_NCs_.

**Figure S4** XRD pattern of Au@Ag_NCs_ nanoparticles, the pattern demonstrating high crystallinity of the silver shell.

**Figure S5** UV vis absorption spectra of Au_NCs_ and Au@Ag_NCs_, indicating LSPR mode tuning and interfacial coupling.

**Figure S6** SERS spectra of PS microplastics at various concentrations using Au@Ag_NCs_.

**Figure S7** Stability evaluation of Au@Ag_NCs_ detection signals(n=50).

**Figure S8** TEM image of Au@Ag_NCs_ adsorbed onto PS microplastic surfaces.

**Figure S9** Raman spectra before and after modification, showing disappearance of the CTAC characteristic peak.

**Figure S10** SERS spectra of PS microplastics (100 μg·mL⁻¹) using Au@Ag_PNCs_ with different aggregation-inducing ions (Zn²⁺, Na⁺, Mg²⁺, Ca²⁺).

**Figure S11** Average particle size distribution of PS microplastics and Au@Ag_PNCs_ before and after Al^3+^ addition(n=3,means±SD).

**Figure S12** Zeta potential of CA filter paper before and after modification with 1 mL of 10 mM dilute hydrochloric acid (HCl) solution.

**Figure S13** Contact angle images of CA filter paper (top: original CA filter paper; bottom: CA filter paper after nanoparticle enrichment).

**Figure S14** (A) XPS survey spectrum of the cellulose acetate membrane. (B) XPS survey spectrum of F_CA_-Au@Ag_NCs_. (C) XPS survey spectrum of F_CA_-Au@Ag_PNC_s (D-F) Ag 3d, O 1s, and S 2p XPS spectra of Ag@Au_PNCs_ enriched on CA filter paper.

**Figure S15** SEM image of CA filter paper(0.1μm), with red box indicating a magnified section.

**Figure S16** Scanning electron microscopy (SEM) images of CA filter paper with different pore sizes (5μm, 1.2μm, 0.45μm, 0.22μm): (i) Red circles indicate magnified regions; (ii) SEN images showing Au@Ag_PNCs_ enrichment efficiency across different pore sizes.

**Figure S17** SERS spectra of PS microplastics (100 μg·mL⁻¹) enriched on CA filter paper with different pore sizes (0.22–5 μm) using Au@Ag_PNCs_.

**Figure S18** (A) SERS spectra of PS microplastics at various concentrations on the F_CA_-Au@Ag_PNCs_ layer. (B) Raman mapping of PS microplastics on the F_CA_-Au@Ag_PNCs_ layer (10μm×10μm). (C) SERS spectra of PS, PMMA, PC, and PVC microplastics obtained using F_CA_-Au@Ag_PNCs_.

**Figure S19** SERS spectra of PS microplastics at various concentrations on the F-F_CA_-Au@Ag_PNCs_ layer.

**Figure S20** (A) Raman mapping of PS microplastics on the F-F_CA_-Au@Ag_PNCs_ layer (10μm×10μm). (B) Bar chart showing stability of PS microplastic detection across seven consecutive batches (n=3, mean±SD).

**Figure S21** (A) SERS spectra of PMMA at different concentrations. (B) SERS spectra of PVC at different concentrations. (C) SERS spectra of PC at different concentrations.

**Figure S22** SERS mapping images of different microplastics on the F-F_CA_-Au@Ag_PNCs_ substrates at their corresponding LDC levels. (A) PMMA, (B) PVC, and (C) PC microplastics. The mapping area was 10 μm × 10 μm for each measurement (10μm×10μm).

**Figure S23** Bar plot of 5-fold cross-validation results for the PLS-DA model, with an average accuracy of 0.9243, standard deviation of 0.0305, and 95% confidence interval of ±0.0611.

**Figure S24** (A) SERS spectra of PS:PMMA mixtures with different composition ratios; (B) SERS spectra of PS:PC mixtures with different composition ratios; (C) SERS spectra of PS:PVC mixtures with different composition ratios; (D) SERS spectra of PS:PMMA:PVC mixtures with different composition ratios.

**Figure S25** 3D-LDA score plots of binary/ternary mixtures (A: PS: PMMA,B: PS: PC,C: PS: PVC,D: PS: PMMA: PVC) (n=250).

**Figure S26** ROC curves and corresponding AUC values for 3D-LDA classification in multicomponent binary systems (A: PS:PMMA, B: PS:PC, C: PS:PVC). All systems show AUC values above 0.9 (****p < 0.0001).

**Figure S27** Multi component systems (A: PS:PMMA, B: PS:PC, C: PS:PVC) Top 15 feature dimension plot

**Figure S28** Overfitting validation of CNN models for multi-component microplastic systems (A: PS:PMMA, B: PS:PC, C: PS:PVC). The results confirm that the CNN-RF hybrid model exhibits excellent generalization performance without overfitting.

**Figure S29** (A) ROC curves and corresponding AUC values for 3D-LDA in PS:PMMA:PVC systems. (B) Schematic of 5-fold cross validation results. (C) Overfitting validation of CNN models for PS:PMMA:PVC systems. (D) shows bar charts of fold wise accuracy for PS:PMMA: PVC. (E) PS:PMMA:PVC systems of the top 15 feature dimensions.

**Figure S30** Bar plots of fold-wise accuracy from 5-fold cross-validation of the CNN model for binary microplastic systems (A: PS:PMMA, B: PS:PC, C:PS:PVC).

**Figure S31** Schematic diagram of the overall computational workflow for establishing the spectral database and performing similarity analysis.

**Figure S32** (A-D) Trends in various metrics of CNN models during training for multiple component systems (PS: PMMA, PS: PC, PVC:PS, PS: PMMA: PVC). From top to bottom, loss function (loss & val_loss), mean absolute error (mae & val_mae), and custom metrics (python_function & val_python_function).

**Figure S33** (A-D) Box plots of prediction deviation for different system ratios using the 1D-CNN model, demonstrating good conservation of proportions.

**Figure S34** (A-E) SERS characteristic peak intensity plots for four typical microplastics, including PVC, PS, PMMA, and PC in different water systems.( Lake Sayram water, Artificial lake water, Songhua River water, Lake Qinghai water, Bohai Sea water, n=50).

**Supplementary Table**

**Table S1** Comparison of the detection limits in this work with reported microplastic concentrations in aquatic environments.

| **Type of detection** | **Limit of detection** | **System** | **Ref.** |
| --- | --- | --- | --- |
| PS | 1.5-32.0 mg·m^-3^ | Sea water | ^[3]^ |
|  | 50.6 ± 34.7 µg·L^-1^ | Fresh surface water | ^[4]^ |
|  | 381-1356 μg·L^−1^(<100 μm) | Lake water(Taihu Lake) | ^[5]^ |
|  | **50 ng·mL^-1^** | Tap water, Lake water, River, Sea water, Qinghai Lake water, Sayram Lake | **This work** |
| PMMA | 381-1356 μg·L^−1^(<100 μm) | Lake water(Taihu Lake) | ^[5]^ |
|  | 0.3-35 mg·L^−1^ | Marine water | ^[6]^ |
|  | **0.5 μg·mL^-1^** | Tap water, lake water, River, Sea water, Qinghai Lake water, Sayram Lake | **This work** |
| PVC | 1.5-32.0 mg·m^-3^ | Sea water | ^[3]^ |
|  | 381-1356 μg·L^−1^(<100 μm) | Lake water(Taihu Lake) | ^[5]^ |
|  | 0.3-35 mg·L^−1^ | Marine water | ^[6]^ |
|  | **1 μg·mL^-1^** | Tap water, lake water, River, Sea water, Qinghai Lake water, Sayram Lake | **This work** |
| PC | 0.206 μg·mL^−1^ | Sea water | ^[7]^ |
|  | 0.70–8400 μg/g (median of 5.9 μg/g) | sewage | ^[8]^ |
|  | **0.5 μg·mL^-1^** | Tap water, lake water, River, Sea water, Qinghai Lake water, Sayram Lake | **This work** |

**Table S2** Comparison of our method with the conventional SERS method for microplastics detection.

| SERS Substrates | Limit of detection | System | Type of detection | Ref. |
| --- | --- | --- | --- | --- |
| AgNPs | 6.25 μg·mL^-1^ | H_2_O, Lake water | PS | ^[9]^ |
| Ag filter membrane | 4.90 mg·L^-1^ | Pond water, tap water, mineral water | PS,PET,PE | ^[10]^ |
| Silver nanoparticles | 5 μg·mL^-1^ | Mineral water, rain water | PS | ^[11]^ |
| AuNP@Cu NPoF | 1 μg·mL^-1^ | dH_2_O | PS | ^[12]^ |
| AgNCAs | 0.001 mg·mL^-1^ | River water, DI water, rain water | PS NPP | ^[13]^ |
| Fe_3_O_4_@PDA-HDTMS, F@PH | 5 μg·mL^-1^ | simulated seawater, tap water, tissue suspensions | PS, PE,PP | ^[14]^ |
| Klarite | 26.25 μg·mL^-1^ | atmosphere | PS,PMMA | ^[15]^ |
| AuNSs@Ag@AAO | 0.05 mg·mL^-1^ | Sea water, Tap water, River water | PS | ^[16]^ |
| AuNP | 0.1 g·L^-1^ | Ultrapure water, Tap water, pond water | PET | ^[17]^ |
| AgNWs/RC film | 0.1 mg·mL^-1^ | DI H_2_O | PS | ^[18]^ |
| Dual-enrichment SERS platform | 50 ng·mL^-1^ | Tap water, lake water, river, seawater, Qinghai Lake water, Sayram Lake | PS; PMMA; PVC; PC | This work |

**Table S3** SERS peak assignments for microplastics

| Raman Shift /cm^-1^ | Assignment | soure | Ref. |
| --- | --- | --- | --- |
| 600 | C-C(O)O stretching | Ester group | ^[19-22]^ |
| 636 | C-Cl stretching | C-Cl bond | ^[23-26]^ |
| 637 | phenyl ring vibration | Benzene ring | ^[20, 27]^ |
| 696 | C-Cl stretching | C-Cl bond | ^[24-26, 28, 29]^ |
| 813 | methylene C–H_2_ stretching vibrations | Methylene | ^[19, 21, 22, 30]^ |
| 887 | O–C(O)–O stretching | Carbonyl group | ^[27]^ |
| 1000 | C–C ring breathing vibrations | Benzene ring | ^[11, 24, 25, 29, 31]^ |
| 1030 | C–H in-plane deformation mode | Benzene ring | ^[25, 32, 33]^ |
| 1111 | C–O–C stretching | Phenolic hydroxyl | ^[20, 27]^ |
| 1238 | C–O–C stretching | Phenolic hydroxyl | ^[20, 27]^ |
| 1430 | CH_2_ bending | Methylene | ^[25, 28, 34]^ |
| 1450 | C-H stretching | Methyl | ^[21, 22]^ |
| 1601 | C–C stretching | Benzene ring | ^[35, 36]^ |
| 1603 | phenyl ring vibration/ C–C stretching | Benzene ring | ^[26, 27]^ |
| 1725 | C=O stretching | Ester group | ^[19-22]^ |

**Table S4** Classification performance metrics of CNN models for binary and ternary microplastic mixtures

|  | Accuracy | Precision | Recall | F1 Score |
| --- | --- | --- | --- | --- |
| PS:PMMA | 0.936 | 0.9841 | 0.984 | 0.984 |
| PS:PC | 0.9 | 0.9756 | 0.975 | 0.9751 |
| PS:PVC | 0.9639 | 0.991 | 0.991 | 0.991 |
| PS:PMMA:PVC | 0.9518 | 0.9881 | 0.9879 | 0.988 |

**TableS5** 5-fold cross-validation performance metrics for binary and ternary microplastic mixtures

|  | Average accuracy rate | standard deviation | 95% fluctuation range |
| --- | --- | --- | --- |
| PS:PMMA | 0.993 | 0.0084 | ±0.0167 |
| PS:PC | 0.923 | 0.0196 | ±0.0391 |
| PS:PVC | 0.98 | 0.0094 | ±0.0187 |
| PS:PMMA:PVC | 0.963 | 0.0144 | ±0.0288 |

**Table S6** MAE values of binary microplastic mixtures from Euclidean Distance and cosine similarity analysis

| ED | | | | | |
| --- | --- | --- | --- | --- | --- |
| PS MAE | 0.343 | PS MAE | 0.2926 | PS MAE | 0.354 |
| PMMA MAE | 0.2941 | PC MAE | 0.3783 | PVC MAE | 0.3812 |
| Cosine | | | | | |
| PS MAE | 0.4058 | PS MAE | 0.362 | PS MAE | 0.4674 |
| PMMA MAE | 0.3483 | PC MAE | 0.3239 | PVC MAE | 0.4898 |

**Table S7** MAE values of ternary microplastic mixtures from Euclidean Distance and cosine similarity analysis

| ED | PS MAE | PMMA MAE | PVC MAE |
| --- | --- | --- | --- |
|  | 0.1739 | 0.0856 | 0.1469 |
| Cosine | PS MAE | PMMA MAE | PVC MAE |
|  | 0.5538 | 0.5043 | 0.4850 |

**Table S8** Adaptive Learning Improves MAE and R² in Binary Polymer CNN Models

| PS MAE | 0.0479 | PS MAE | 0.0936 | PS MAE | 0.0722 |
| --- | --- | --- | --- | --- | --- |
| PMMA MAE | 0.0494 | PC MAE | 0.0979 | PVC MAE | 0.0733 |
| PS R^2^ | 0.9625 | PS R^2^ | 0.8541 | PS R^2^ | 0.9115 |
| PMMA R^2^ | 0.9627 | PC R^2^ | 0.8263 | PVC R^2^ | 0.8935 |

**Table S9** Adaptive Learning Improves MAE and R² in Ternary Polymer CNN Models

| PS MAE | PMMA MAE | PVC MAE |
| --- | --- | --- |
| 0.0296 | 0.0443 | 0.0368 |
| PS R^2^ | PMMA R^2^ | PVC R^2^ |
| 0.9092 | 0.9392 | 0.8435 |

**Supplementary Text**

**Text S1 Additional characterization, including HRTEM, DLS, XRD, and UV–vis spectroscopy**

TEM and SEM results (**Figures 2B** and **S1**) show that the particles have a regular cubic shape, demonstrating excellent uniformity and controllability. The DLS results further confirm a narrow hydrodynamic size distribution with an average hydrodynamic diameter of 74.19 ± 0.42 nm (mean ± standard deviation, n = 3; RSD = 0.57%), indicating good reproducibility of the synthesis process. HRTEM analysis revealed highly crystalline lattice fringes in the shell region, with spacings of 0.235 nm and 0.203 nm corresponding to Ag (111) and Ag (200), respectively, supporting the formation of a highly crystalline face-centered cubic Ag shell favorable for plasmonic enhancement (**Figure S3**). EDS elemental mapping (**Figure S2**) shows Ag evenly distributed in the outer layer while Au is concentrated in the core, further confirming the typical core shell structure. XRD peaks at 38.2° and 44.4° are consistent with the fcc structure of noble metals and are close to the Ag reference pattern (**Figure S4**). However, since Au and Ag share similar fcc diffraction properties, the formation of an Ag shell is more directly supported by HRTEM lattice analysis and EDS elemental mapping. The UV-vis spectrum of Au@Ag_NCs_ (**Figure S5**) shows two plasmon-related absorption bands at 403 and 513 nm. The band at 403 nm can be assigned to the Ag-dominated plasmon resonance, while the band at 513 nm is associated with a hybridized Au-core-related plasmon mode. In contrast to pure Au_NCs_, which exhibit a dominant resonance at 532 nm, Au@Ag_NCs_ exhibit a modified plasmonic absorption profile characterized by a blue shifted feature at 513 nm and an additional red shifted shoulder around 605 nm. These spectral variations suggest that the Au@Ag core-shell architecture modifies the fundamental plasmonic response, possibly due to plasmon hybridization between the Au core and the Ag shell. Together with the structural characterization, these results support the successful formation of Au@Ag core–shell nanocubes with altered optical properties.

**Text S2 Determination of membrane retention efficiency for PS microplastics using nanoparticle tracking analysis (NTA)**

To evaluate the retention performance of cellulose acetate (CA) membranes with different pore sizes (5 μm, 1.2 μm, 0.45 μm, 0.22 μm, and 0.1 μm), nanoparticle tracking analysis (NTA) was used to determine the particle number concentrations of polystyrene (PS) microplastic suspensions before and after filtration through Au@Ag_PNC_-functionalized CA membranes.

As shown in **Figure T1,** the initial particle concentration of the 25 μg mL⁻¹ PS suspension was approximately 7.43 × 10⁹ particles mL⁻¹. The concentrations measured after filtration through the 5 μm, 1.2 μm, 0.45 μm, 0.22 μm, and 0.1 μm CA membranes were approximately 7.09 × 10⁹, 6.06 × 10^9^, 3.45 × 10^9^, 1.16 × 10^9^, and 7.03 × 10^8^ particles mL⁻¹, respectively.

The membrane retention rate was calculated according to Equation^[37]^

R (%) = (C_0_ – C_f_) / C_0_ × 100%

where 𝑅 represents the membrane retention rate, and C_0_ and C_f_ epresent the particle number concentrations before and after filtration, respectively.

Based on the NTA measurements, the calculated retention rates for the 5 μm, 1.2 μm, 0.45 μm, 0.22 μm, and 0.1 μm CA membranes were 4.58%, 18.44%, 53.57%, 84.39%, and 90.54%, respectively. These results indicate that the 0.1 μm CA membrane exhibited the highest particle retention efficiency among the tested membranes.

**Figure T1** Figure T1. NTA measurement results of PS microplastic suspensions before and after filtration through CA membranes with different pore sizes: (A) initial PS suspension (25 μg mL⁻¹); (B–F) filtrates collected after filtration through 5 μm, 1.2 μm, 0.45 μm, 0.22 μm, and 0.1 μm CA membranes, respectively.

UV-vis spectroscopy has been used only as an auxiliary qualitative method to monitor the relative changes in the scattering signal of suspended particles under different filtration conditions. The observed scattering trends are in qualitative agreement with the retention behavior determined from the NTA measurements.

**Text S3 Calculation of the Enhancement Factor (EF)**

1. Calculation of the Enhancement Factor (EF) for PS

The enhancement factor (EF) of PS can be calculated as^[38]^:

EF = (I_SERS_ / I_Raman_) × (N_Raman_ / N_SERS_)

I_SERS_ was the peak intensity of the 1000 cm^-1^ peak in the SERS spectrum and I_Raman_ was the peak intensity of the same vibrational mode.

1, Pure PS microplastics were absorbed using a capillary tube with an inner diameter of 0.5 mm from a 25 mg·mL^-1^ PS solution. SERS detection was performed directly under a 10× microscope with a laser diameter of 1298 nm. I_Raman_= 30.512987

2, Au@Ag_PNCs_, Take 5 μL of 0.5 mg·mL^-1^ PS solution and 0.5μL of 10mM Al^3+^ solution and mix it with 4.5 μL of Au@Ag_PNCs_ colloid. Absorb the mixture using a capillary tube with an inner diameter of 0.5 mm. Perform SERS detection under a 10× microscope with a laser diameter of 1298 nm. I_SERS_ = 976.63312

C_SERS_=0.5×5/ (5+0.5+4.5) = 0.25 mg·mL^-1^ =2.5×10^-4^g·mL^-1^

C_Raman_=25mg·mL^-1^=0.025g·mL^-1^

EF = (I_SERS_ × C_Raman_) / (I_Raman_ × C_SERS_) = (976.63312×0.025)/( 30.512987×2.5×10^-4^)=8.002×10^3^

Using the same procedure, the enhancement factor EF for the PS substrate is calculated as 8.002×10^3^.

3, Au@Ag_PNCs_ were enriched on CA filter paper. A 1 mL sample of 0.1 mg·mL^-1^ PS solution was drawn into a syringe and used to filter microplastics onto the paper. SERS detection was performed under 10× magnification with a laser diameter of 1298 nm.I_SERS_= 1633.1628

C_SERS_=0.1mg·mL^-1^ =1×10^-4^g·mL^-1^

C_Raman_=25mg·mL^-1^=0.025g·mL^-1^

EF = (I_SERS_ × C_Raman_) / (I_Raman_ × C_SERS_) = (1633.1628×0.025)/( 30.512987×1×10^-4^)=1.338×10^4^

Using the same procedure, the enhancement factor EF for the PS substrate is 1.338 × 10^4^.

4, Au@Ag_PNCs_ were enriched on CA filter paper. For re-enrichment with Au@Ag_PNCs_ nanoparticles, 1 mL of 10 μg·mL^-1^ PS solution was drawn into a syringe, allowing microplastics to filter onto the paper. Au@Ag_PNCs_ nanoparticles were then drawn into the syringe to enrich them onto the paper. SERS detection was performed under 10× magnification with a laser diameter of 1298 nm. I_SERS_ = 10592.369

C_SERS_=0.01mg·mL^-1^ =1×10^-5^ g·mL^-1^

C_Raman_=25mg·mL^-1^=0.025 g·mL^-1^

EF = (I_SERS_ × C_Raman_) / (I_Raman_ × C_SERS_) = (10592.369×0.025)/(30.512987×1×10^-5^)=8.679×10^5^

Using the same procedure, the enhancement factor EF for the PS substrate is calculated as 8.679×10^5^.

Under identical conditions, record these four spectra using a 532 nm laser with an exposure time set to 10 s for single shot accumulation.

2. Calculation of the Enhancement Factor (EF) for PMMA

1, Pure PMMA microplastics were absorbed using a capillary tube with an inner diameter of 0.5 mm from a 25 mg·mL^-1^ PMMA solution. SERS detection was performed directly under a 10× microscope with a laser diameter of 1298 nm. I_Raman_= 48.193359

2, Au@Ag_PNCs_ were enriched on CA filter paper. For re-enrichment with Au@Ag_PNCs_ nanoparticles, 1 mL of 10 μg·mL^-1^ PMMA solution was drawn into a syringe, allowing microplastics to filter onto the paper. Au@Ag_PNCs_ nanoparticles were then drawn into the syringe to enrich them onto the paper. SERS detection was performed under 10× magnification with a laser diameter of 1298 nm. I_SERS_ = 2187.3801

C_SERS_=0.01mg·mL^-1^ =1×10^-5^ g·mL^-1^

C_Raman_=25mg·mL^-1^=0.025 g·mL^-1^

EF = (I_SERS_ × C_Raman_) / (I_Raman_ × C_SERS_) = (2187.3801×0.025)/( 48.193359×1×10^-5^)=1.135×10^5^

Using the same procedure, the enhancement factor EF for the PMMA substrate is calculated as 1.135×10^5^.

It should be noted that a direct comparison of EF values across different polymers may be affected by variations in their intrinsic Raman cross sections and molecular structures. In this work, representative microplastics were selected as target analytes to evaluate the practical detection performance of the substrate. While the use of a common model analyte could provide a more standardized basis for EF comparisons, the current study focuses on application-oriented evaluation and further systematic normalization using a model analyte will be explored in future work.

Note: Raman measurements were performed using a Raman AFM system (WITec 𝛼300 RA, Germany) with the following parameters, laser wavelength: 532 nm, grating: 600 grooves min^-1^, resolution: 3 cm^-1^, scanning time: 10 s, and laser power: 15 mW, and 1 accumulation.

**Text S4 Evaluation of reproducibility and stability**

The batch-to-batch reproducibility of the SERS platform was systematically evaluated. Ten batches of Au@Ag nanoparticles were consecutively synthesized and used for the detection of polystyrene (PS) microplastics. Statistical analysis of the characteristic peak intensity at 1000 cm⁻¹ yielded a relative standard deviation (RSD) of 4.75%, demonstrating good batch-to-batch consistency and reproducibility of the prepared substrates (**Figure T2A**).

In addition, the stability of the system was evaluated in terms of storage stability and reusability. For storage stability, the same SERS substrate was stored under ambient conditions and measured every two days for a total of seven measurements, with 50 spectra collected at each time point. The results showed an RSD of 3.69% (**Figure T2B**), indicating stable signal responses during the storage period. For reusability, the same CA membrane was reused after being rinsed under running water for repeated detection. The obtained SERS signals exhibited an RSD of 3.93% (**Figure T2C**), suggesting that the functionalized surface maintained its performance without significant degradation during multiple cycles.

Overall, CA membrane-based systems demonstrate good reproducibility, stability, and durability under batch-to-batch variations, storage conditions, and repeated use.

**Figure T2** Bar charts of batch-to-batch reproducibility (A), storage stability (B), and reusability (C) for PS microplastic detection using the proposed method.

**FigureT3** Optical microscopy images and SEM images of PS microplastics before (A–B) and after (C–D) aging.

**Text S5 Microplastic aging and matrix interference assessment**

To assess the impact of environmental factors on detection performance, microplastic aging and matrix interference experiments were performed. PS microplastics were subjected to accelerated aging under alternating UV irradiation (120 W, 365 nm) and elevated temperature (72 °C) (**Figure T3**). The results show that the characteristic peak at 1000 cm⁻¹ remained stable during the aging process, indicating that aging has limited impact on the main Raman features **(Figure T4**).

In addition, matrix interference was evaluated by introducing dyes (R6G, crystal violet) and metal ions (Cu²⁺, Fe²⁺, Zn²⁺) into PS samples (**Figure T5**). Only the characteristic signal of microplastics is observed in the SERS spectra, with no detectable signal from intervening species. This is attributed to the pressure-assisted filtration process, which enriches the microplastics while removing the dissolved interferents.

Overall, the method is applicable, to some extent, to the detection of aged microplastics and shows resistance to interference from commonly dissolved matrix.

**Figure T4** SERS spectra of PS microplastics at different aging times.

**Figure T5** SERS spectra of PS microplastic samples with the addition of metal ions (Cu^2+^, Fe^2+^, and Zn^2+^) (A) and dyes (Rhodamine 6G and Crystal Violet) (B).

**Text S6 Qualitative analysis and data processing workflow**

In this study, SERS measurements were performed using substrates synthesized from five independent batches. Both binary systems (PS:PMMA, PS:PC, and PVC:PS) and a ternary system (PS, PMMA, and PVC) were investigated. For the binary systems, five mixing ratios (1:9, 3:7, 5:5, 7:3, and 9:1) were prepared, while for the ternary system, five compositions (2:2:6, 2:6:2, 4:2:4, 4:4:2, and 6:2:2) were designed. For each composition, 250 SERS spectra were collected across 10 temporal batches (25 spectra per batch), resulting in a total of 1,250 spectra for both the binary and ternary systems, which were used for subsequent model analysis.

The dataset was divided into a training set (80%) and a test set (20%). The two sets are strictly separated and normalized independently to prevent data leakage.

Stratified sampling were employed to randomly partition the training set into five equal and non-overlapping subsets, ensuring a consistent data distribution across all subsets. Based on this, five-fold cross-validation is performed, where four subsets are used for model training and the remaining subset is used for validation, iteratively repeated five times.

In addition, for external testing, newly synthesized substrates from separate batches and separately prepared samples are used to ensure that the test data is completely independent of the training process^[39]^.

**Reference**

[1] Li, R.; Hu, Y.; Sun, X.; Zhang, Z.; Chen, K.; Huang, S.; Liu, S.; Liu, Q.; Chen, X. Machine Learning-Aided 3D Dynamic SERS Strategy for Physiological Mapping: Biotoxicity of Environmentally Dimensional Aged Nanoplastics and Corresponding Protein Corona Complexes. *Anal. Chem.,* **2024**, *96* (42), 16629-16638.

[2] Li, R.; Sun, X.; Hu, Y.; Liu, S.; Huang, S.; Zhang, Z.; Chen, K.; Liu, Q.; Chen, X. Machine Learning-Assisted “Shrink-Restricted” SERS Strategy for Classification of Environmental Nanoplastic-Induced Cell Death. *Environ. Sci. Technol.,* **2024**, *58* (51), 22528-22538.

[3] ten Hietbrink, S.; Materić, D.; Holzinger, R.; Groeskamp, S.; Niemann, H. Nanoplastic concentrations across the North Atlantic. *Nature,* **2025**, *643* (8071), 412-416.

[4] Sefiloglu, F. Ö.; Brits, M.; van Velzen, M. J. M.; Pieke, E. N.; Kramer, O. J. I.; Lamoree, M. H. Microplastics in drinking water: quantitative analysis of microplastics from source to tap by pyrolysis–gas chromatography-mass spectrometry. *Environ. Sci. Pollut. Res.,* **2025**, *32* (45), 25603-25617.

[5] Li, Q.; Bai, Q.; Zheng, R.; Li, P.; Liu, R.; Yu, S.; Liu, J. Mass concentration, spatial distribution, and risk assessment of small microplastics (1–100 μm) and nanoplastics (<1 μm) in the surface water of Taihu Lake, China. *Environ. Res.,* **2025**, *285*, 122214.

[6] Barkhau, J.; Sanchez, A.; Lenz, M.; Thiel, M. Effects of microplastics (PVC, PMMA) on the mussel Semimytilus algosus differ only at high concentrations from those of natural microparticles (clay, celite). *Mar. Pollut. Bull.,* **2022**, *177*, 113414.

[7] Chen, F.; Zhao, M.; Zhang, B.; Li, G.; Liu, H.; Li, Z.; Zhao, M.; Ma, Y. Gas-liquid interface plasmonic arrays for SERS detection of microplastics. *Appl. Surf. Sci.,* **2025**, *690*, 162583.

[8] Zhang, J.; Wang, L.; Halden, R. U.; Kannan, K. Polyethylene Terephthalate and Polycarbonate Microplastics in Sewage Sludge Collected from the United States. *Environ. Sci. Technol. Lett.,* **2019**, *6* (11), 650-655.

[9] Hu, R.; Zhang, K.; Wang, W.; Wei, L.; Lai, Y. Quantitative and sensitive analysis of polystyrene nanoplastics down to 50 nm by surface-enhanced Raman spectroscopy in water. *J. Hazard. Mater.,* **2022**, *429*, 128388.

[10] Wu, J.; Li, Y.; Liu, X.; Fang, B.; Wu, D.; Wang, Q.; Gong, Z.; Wu, Y. Integration of bifunctional silver dendrite membranes with surface-enhanced Raman scattering for sensitive detection of polystyrene microplastics in aquatic environments. *J. Hazard. Mater.,* **2024**, *480*, 136394.

[11] Ruan, X.; Xie, L.; Liu, J.; Ge, Q.; Liu, Y.; Li, K.; You, W.; Huang, T.; Zhang, L. Rapid detection of nanoplastics down to 20 nm in water by surface-enhanced raman spectroscopy. *J. Hazard. Mater.,* **2024**, *462*, 132702.

[12] Pant, U.; Tate, J.; Liu, X.; Birse, N.; Elliott, C.; Cao, C. From automated Raman to cost-effective nanoparticle-on-film (NPoF) SERS spectroscopy: A combined approach for assessing micro- and nanoplastics released into the oral cavity from chewing gum. *J. Hazard. Mater.,* **2025**, *486*, 136978.

[13] Liu, Y.; Qin, Z.; Wang, G.; Jia, X.; Zhou, J.; Li, H.; Li, Z.; Fang, X. Size-dependent selectivity and quantification on detecting PS nanoplastics particles in a mixed solution with different diameters by using periodic Ag nanocavities SERS substrates with high sensitivity. *J. Hazard. Mater.,* **2025**, *485*, 136943.

[14] Xu, H.; Jin, Y.; Dong, C.; Cheng, J.; Yu, Z.; Chen, C.; Ma, D.; Ji, W.; Xie, Y. Magneto-Photothermal Synergistic Hydrophobicity Nanoplatform for Efficient Enrichment and Ultrasensitive Detection of Micro-Nanoplastics. *Environ. Sci. Technol.,* **2025**, *59* (49), 26396-26407.

[15] Xu, G.; Cheng, H.; Jones, R.; Feng, Y.; Gong, K.; Li, K.; Fang, X.; Tahir, M. A.; Valev, V. K.; Zhang, L. Surface-Enhanced Raman Spectroscopy Facilitates the Detection of Microplastics <1 μm in the Environment. *Environ. Sci. Technol.,* **2020**, *54* (24), 15594-15603.

[16] Lê, Q. T.; Ly, N. H.; Kim, M.-K.; Lim, S. H.; Son, S. J.; Zoh, K.-D.; Joo, S.-W. Nanostructured Raman substrates for the sensitive detection of submicrometer-sized plastic pollutants in water. *J. Hazard. Mater.,* **2021**, *402*, 123499.

[17] Xu, D.; Su, W.; Lu, H.; Luo, Y.; Yi, T.; Wu, J.; Wu, H.; Yin, C.; Chen, B. A gold nanoparticle doped flexible substrate for microplastics SERS detection. *Phys. Chem. Chem. Phys.,* **2022**, *24* (19), 12036-12042.

[18] Jeon, Y.; Kim, D.; Kwon, G.; Lee, K.; Oh, C.-S.; Kim, U.-J.; You, J. Detection of nanoplastics based on surface-enhanced Raman scattering with silver nanowire arrays on regenerated cellulose films. *Carbohydr. Polym.,* **2021**, *272*, 118470.

[19] Li, G.; Yang, Z.; Pei, Z.; Li, Y.; Yang, R.; Liang, Y.; Zhang, Q.; Jiang, G. Single-particle analysis of micro/nanoplastics by SEM-Raman technique. *Talanta,* **2022**, *249*, 123701.

[20] Anger, P. M.; von der Esch, E.; Baumann, T.; Elsner, M.; Niessner, R.; Ivleva, N. P. Raman microspectroscopy as a tool for microplastic particle analysis. *TrAC, Trends Anal. Chem.,* **2018**, *109*, 214-226.

[21] Zhao, M.; Sikdar, D.; Zhao, M.; Ma, Y. 3D Self-Assembly of a Bilayer Nanoparticle Metasurface for Surface-Enhanced Raman Scattering (SERS) Sensing. *Nano Lett.,* **2025**, *25* (20), 8251-8257.

[22] Xie, L.; Gong, K.; Liu, Y.; Zhang, L. Strategies and Challenges of Identifying Nanoplastics in Environment by Surface-Enhanced Raman Spectroscopy. *Environ. Sci. Technol.,* **2023**, *57* (1), 25-43.

[23] Ayeni, O.; Shondo, J. N.; Tjardts, T.; Şen, S.; Faupel, F.; Veziroglu, S.; Aktas, O. C.; Ameri, T. Photo-induced Raman amplification for detecting and monitoring degradation of micro- and nano-sized PVC plastics. *J. Mater. Chem. A,* **2025**.

[24] Hong, Z.; Zhang, Y.; Duan, F.; Du, J.; Sun, Z. Flexible Au tape-based SERS sensor for atmospheric microplastic detection. *Environmental Chemistry and Ecotoxicology,* **2025**, *7*, 1798-1805.

[25] Xie, L.; Luo, S.; Liu, Y.; Ruan, X.; Gong, K.; Ge, Q.; Li, K.; Valev, V. K.; Liu, G.; Zhang, L. Automatic Identification of Individual Nanoplastics by Raman Spectroscopy Based on Machine Learning. *Environ. Sci. Technol.,* **2023**, *57* (46), 18203-18214.

[26] Liu, Y.; Jiang, W.-Y.; Liao, Y.; Sun, R.; Hu, J.; Lu, Z.; Chang, M.; Yang, J.; Dai, Z.; Zhou, C.; Hong, P.; Qian, Z.-J.; Sun, S.; Ren, L.; Liang, Y.-Q.; Zhang, Y.; Li, C. Separation of false-positive microplastics and analysis of microplastics via a two-phase system combined with confocal Raman spectroscopy. *J. Hazard. Mater.,* **2022**, *440*, 129803.

[27] Jin, N.; Song, Y.; Ma, R.; Li, J.; Li, G.; Zhang, D. Characterization and identification of microplastics using Raman spectroscopy coupled with multivariate analysis. *Anal. Chim. Acta,* **2022**, *1197*, 339519.

[28] Jung, E. S.; Choe, J. H.; Kim, J. S.; Ahn, D. W.; Yoo, J.; Choi, T. M.; Pyo, S. G. Quantitative Raman analysis of microplastics in water using peak area ratios for concentration determination. *npj Clean Water,* **2024**, *7* (1), 104.

[29] Sobhani, Z.; Al Amin, M.; Naidu, R.; Megharaj, M.; Fang, C. Identification and visualisation of microplastics by Raman mapping. *Anal. Chim. Acta,* **2019**, *1077*, 191-199.

[30] Wang, Y.; Yu, C.; Ji, H.; Liu, Z.; Wang, X.; Ji, Y.; Sun, X.; Zhao, Y.; Qiu, X.; Zhang, T.; Li, J.; Liu, X.; Lv, X.; Cai, B.; Zhao, Y.; Huang, J.-A.; Li, Y. Label-free therapeutic drug monitoring in human serum by the 3-step surface enhanced Raman spectroscopy and multivariate analysis. *Chem. Eng. J.,* **2023**, *452*, 139588.

[31] Seo, D.; Lim, D.; Seo, J.; Shin, D. Quantification of auto-photobleaching effects during Raman measurements for microplastic detection. *Sens. Actuators B Chem.,* **2025**, *423*, 136702.

[32] Caponigro, V.; Di Fiore, C.; Carriera, F.; Iannone, A.; Malinconico, A.; Campiglia, P.; Crescenzi, C.; Avino, P. Evaluating microplastic emission from takeaway containers: A Micro-Raman approach across diverse exposure scenarios. *Food Chem.,* **2025**, *464*, 141716.

[33] Kim, J. Y.; Koh, E. H.; Yang, J.-Y.; Mun, C.; Lee, S.; Lee, H.; Kim, J.; Park, S.-G.; Kang, M.; Kim, D.-H.; Jung, H. S. 3D Plasmonic Gold Nanopocket Structure for SERS Machine Learning-Based Microplastic Detection. *Adv. Funct. Mater.,* **2024**, *34* (2), 2307584.

[34] Luo, Y.; Al Amin, M.; Gibson, C. T.; Chuah, C.; Tang, Y.; Naidu, R.; Fang, C. Raman imaging of microplastics and nanoplastics generated by cutting PVC pipe. *Environ. Pollut.,* **2022**, *298*, 118857.

[35] Krekelbergh, N.; Li, J.; Kusumawardani, P. N.; Liu, Y.; Hu, J.; Sleutel, S.; Parakhonskiy, B.; Hoogenboom, R.; De Neve, S.; Skirtach, A. Comparison of Raman and fluorescence microscopy for identification of small (< 2 μm) microplastics in soil. *Environ. Pollut.,* **2025**, *374*, 126204.

[36] Liu, Y.; Wei, Y.; Wang, G.; Jia, X.; Zhou, J.; Li, H.; Chang, H.; Li, Z.; Jin, Z.; Li, K. Reliable detection and identification of micro and nano plastics via convolutional neural network-driven SERS technology. *J. Environ. Chem. Eng.,* **2025**, *13* (5), 118297.

[37] Guo, X.; Li, S.; Wang, T.; Su, J.; Liu, Y.; Chen, J.; Zhan, J. Systematic quantitation for microplastics and nanoplastics based on size-fractionated filtration hyphenated to Raman/SERS and slope-matching strategy. *J. Hazard. Mater.,* **2025**, *494*, 138488.

[38] Wang, Y.; Ma, C.; Zhao, X.; Li, J.; Han, J.; Lv, X.; Li, G.; Bi, Y.; Li, Y. Self-Assembled Hydrophobic Confinement Coupled with Calcium Coordination Enables Selective and Label-Free SERS Detection of Urinary Biomarkers in Complex Samples. *Adv. Funct. Mater.,* **2026**, *36 (6),* e14570.

[39] Kang, Z.; Li, Y.; Liu, J.; Chen, C.; Wu, W.; Chen, C.; Lv, X.; Liang, F. H-CNN combined with tissue Raman spectroscopy for cervical cancer detection. *Spectrochim. Acta. A Mol. Biomol. Spectrosc.,* **2023**, *291*, 122339.
